# Interpeduncular GABAergic neuron function controls threat processing and innate defensive adaptive learning

**DOI:** 10.1038/s41380-025-03131-9

**Published:** 2025-08-08

**Authors:** Elora W. Williams, Leshia Snively, Benjamin R. O’Meara, Hannah L. Jacobs, Miranda Kolb, Rubing Zhao-Shea, Rebecca G. Pavchinskiy, Emma Keppler, Michael V. Baratta, Andrew R. Tapper, Susanna Molas

**Affiliations:** 1 Institute for Behavioral Genetics, University of Colorado Boulder, Boulder, CO USA; 2 Department of Psychology and Neuroscience, University of Colorado Boulder, Boulder, CO USA; 3 Department of Neurobiology, Brudnick Neuropsychiatric Research Institute, University of Massachusetts Chan Medical School, Worcester, MA USA; 4 Crnic Institute Boulder Branch, BioFrontiers Institute, University of Colorado Boulder, Boulder, CO USA

**Keywords:** Neuroscience, Psychology, Psychiatric disorders

## Abstract

The selection of appropriate defensive behaviors in the face of potential threat is fundamental to survival. Equally important is learning to adjust and optimize defensive behaviors when there is no real danger. Despite extensive research on innate threat processing, little is known about the mechanisms by which individuals adapt their defensive behaviors in response to repeated threats that lack real danger. Insight into this process is critical as its dysregulation may contribute to neuropsychiatric conditions, such as anxiety disorders. Here, we used the visual looming stimulus (VLS) paradigm in mice to investigate innate threat processing and adaptive defensive learning. Repeated exposure to VLS over consecutive sessions reduced immediate freezing responses and time spent inside a sheltered area upon VLS events, leading to an increase in exploratory behaviors. Fiber photometry recordings and optogenetic manipulations revealed that VLS innate adaptive defensive learning is associated with reduced recruitment of the midbrain interpeduncular nucleus (IPN), a structure associated with fear and anxiety-related behaviors. Functional circuit-mapping identified a role for select IPN projections to the laterodorsal tegmental nucleus in gating defensive learning. Finally, we uncovered a subpopulation of IPN neurons that express the neuropeptide somatostatin and encode avoidance signals in response to VLS. These results identify critical behavioral signatures of innate defensive responses and a circuit that regulates the essential features of threat processing.

## Introduction

Individuals select optimal defensive strategies, such as escape or freezing, to avoid danger when threat is present [1, 2]. Nevertheless, with repeated exposures to a potential threat without evidence of an aversive outcome, defensive behaviors must undergo adaptive learning, thus contributing to efficient action selection. Numerous neuropsychiatric conditions, such as anxiety disorders, manifest maladaptation of threat responses [3], highlighting the importance of understanding these basic neurobehavioral processes.

Exposure to an overhead dark visual looming stimulus (VLS) naturally elicits innate defensive responses across multiple species [1, 4]. In rodents, the detection of an expanding VLS to the upper visual field triggers a sequence of stimulus-evoked responses, starting with immediate freezing followed by escaping towards a sheltered area, and ending with shelter-based avoidance [5]. However, how rodents optimize these defensive sequences with repeated VLS and the mechanisms that support this innate defensive adaptive learning remain largely unknown.

Processing sensory information and coordinating appropriate motor output for defensive behaviors requires complex neural circuits [6]. Retinal neurons detect visual threats and rapidly convey threat signals to the superior colliculus (SC), the lateral geniculate nucleus or the dorsal raphe nucleus to generate freezing and escape responses [7–9]. Additional circuits, including the amygdala and hypothalamic nucleus, converge inputs in the periaqueductal gray (PAG) to guide motor outputs [6]. Rodent and human studies have implicated an emerging circuit, arising in the habenula, that contributes to threat-related behaviors [10]. The habenula nuclei play a crucial role in regulating emotional, motivational, and cognitive behaviors [11–14], with the medial part of the habenula (mHb) sending descending projections almost exclusively to the interpeduncular nucleus (IPN) of the midbrain [15], an axis implicated in anxiety [16–23] and fear responses [24–27]. As compared to other regions traditionally involved in aversive processing, such as the amygdala, the IPN represents an important brain center that amplifies responses to aversive stimuli [28]. The IPN is highly enriched in GABAergic neurons that establish reciprocal connections with regions involved in motivation and affective behaviors, including the raphe, locus coeruleus, nucleus incertus (NI) and laterodorsal tegmental nucleus (LDTg) [29, 30]. Recent work indicates that the habenulo-interpeduncular axis, and IPN projections to the LDTg, mediate aversive and reward-related behaviors [31, 32]. Yet, the extent to which VLS-driven recruitment of the IPN and its circuits facilitate adaptive changes in defensive behaviors is still unclear.

## Materials and methods

### Animals

All experiments followed guidelines provided by the National Research Council with approved animal protocols from the Institutional Animal Care and Use Committee of the University of Massachusetts Chan Medical School. C57Bl/6J (Stock #000664, Jackson), *GAD2*^*Cre*^ (Stock #10802, Jackson) and *Sst*^*Cre*^ (Stock #013044, Jackson) mice were used. *Cre* lines were crossed with C57Bl/6J mice and heterozygous animals were used. All experiments included male mice. IPN Sst ablation experiments and c-Fos data included males and females. Subject mice were kept under a reverse 12 h light/dark cycle (lights ON at 7 P.M.) for 3–4 weeks with *ad libitum* access to food and water, and individually housed for at least 5 days before behavioral testing (9–14 weeks old). Experiments were performed during the dark cycle (8 A.M.–5 P.M.).

### Viral preparations

Biosensors, optogenetic and control plasmids packaged into viral particles were purchased from Addgene. For fiber photometry experiments we used pAAV.CAG.Flex.GCaMP6m.WPRE.SV40 (#100839-AAV5, 2.6 × 10^13^GC/ml), pGP.AAV.CAG.Flex.-jGCaMP7s.WPRE (#104495-AAVrg, 1.1 × 10^13^GC/ml). For tracing and optogenetic experiments we used pAAV.hSyn.DIO.EGFP (#50457-AAV5, 1.3 × 10^13^GC/ml and -AAVrg, 1.4 × 10^13^GC/ml), pAAV.hSyn.DIO.mCherry (#50459-AAV5, 1.8 × 10^13^GC/ml), pAAV.hSyn.mCherry (#114472-AAV2, 2.6 × 1013 GC/ml), pAAV.Ef1a.DIO.eNpHR3.0.EYFP (#26966-AAV5, 3.8 × 10^12^GC/ml), pAAV.Ef1a.doublefloxed.hChR2(H134R).mCherry.WPRE.HGHpA (#20297-AAV5 1.2 × 10^13^GC/ml) and pAAV.flex.taCasp3-TEVp (#45580-AAV5, 2.5 × 10^13^GC/ml). The viral stock pAAV.Ef1a.DIO.-eNpHR3.0.EYFP (#AV9115-rAAV2, 5.8 × 10^12^VM/ml) was obtained from UNC GTC Vector Core.

### Stereotaxic surgeries

Surgeries were performed under aseptic conditions as previously described [23]. Mice (6–8 weeks old) were deeply anaesthetized using 100 mg/kg ketamine (VEDCO) and 10 mg/kg xylazine (LLOYD) and placed on a stereotaxic frame (Stoelting Co.). Viral solutions were microinjected at a controlled rate of 50 nl/min using a gas-tight 33 G 10 μl neurosyringe (1701RN; Hamilton). Injection coordinates were (in mm, anteroposterior, mediolateral, dorsoventral and angle): IPN (−3.4, −0.5, −4.86, 6°) and LDTg (−5.34, ±0.4, −3.2, 0°). Viral volumes were 300 nl (IPN) and 300 nl/side (LDTg). For fiber photometry and optogenetic experiments, 3–5 weeks post-viral injection, an optic fiber implant (200 μm core diameter; 0.53 N.A., Doric Lenses) held in a magnetic aluminum receptacle (Doric Lenses) was placed above the IPN and secured into the skull using adhesive (C&B Metabond cement, Parkell Inc.) followed by dental cement (Cerebond, PlasticsOne). Mice received IP injections of 1 mg/kg ketoprofen analgesic (Zoetis) and monitored for recovery. Mice were randomly assigned to experimental groups. Animals showing no virus, off-target viral expression, or incorrect optic fiber placement (<10%) were excluded from analysis.

### Behavioral experiments

#### Visual looming stimulus (VLS) paradigm

The apparatus consisted of a rectangular Plexiglass maze (40 × 22 × 30 cm) with a projector screen (30 × 20 cm) above the arena and a rectangular shelter (10 × 12 cm) in one corner. All mice habituated to the apparatus for 8–10 min. 24 h later, after 2–5 min, a VLS was randomly displayed from the screen while mice actively explored the arena. Each VLS consisted of 15 consecutive 0.5 s dark expansions and each mouse received 4–7 looming trials/day with a minimum 60 s inter-looming trial interval. The VLS test session was repeated for 3 consecutive days. For side-VLS, the screen was displayed from a wall view. The apparatus was cleaned between animals with 0.1% Micro-90 solution. A video-camera was used to record and track animal behavior using Ethovision XT (v15.0). The arena was subdivided into a nest area (12 × 10 cm), safety zone adjacent to the nest (10 × 10 cm), trigger zone where the VLS were displayed (12 × 12 cm) and zone near the walls (5 cm). Immediate freezing and maximum speed were reported 2 s upon VLS initiation. Escape run was considered as maximum speed 10 s upon VLS. The percentage of time in each zone was estimated within 30 s upon VLS initiation and averaged for each animal. Latency to and time in nest were manually scored by an experimenter blind to animal conditions.

#### Foot shock

Mice were habituated to a fear conditioning cage. GCaMP fluorescence from IPN neurons was recorded for 2 min before the first shock. During a 15 min foot-shock period, ten shocks (0.5 mA, 1 s duration) were delivered at random intervals and time-stamped into the photometry recording via a transistor-transistor logic (TTL) pulse from the fear conditioning system.

#### Open field

The apparatus consisted of an open-field chamber (42 × 38 × 30 cm). Each mouse was given 10 min to explore, and the time spent in the center and outer parts of the chamber was tracked from a video recording using Ethovision XT.

#### Elevated plus maze

The elevated plus maze (EPM) consisted of a central junction (5 × 5 cm) and four arms elevated 45 cm above the floor with each arm positioned at 90° relative to the adjacent arms. Two closed arms were enclosed by high walls (30 × 5 × 15 cm) and open arms were exposed (30 × 5 × 0.25 cm). Mice were given 5 min of free exploration.

#### Tail lift

Animals were picked up by their tails by an experimenter while they were actively exploring the home cage.

### Fiber photometry and data analysis

GCaMP signals were recorded using a Doric Instruments Fiber Photometry System as previously described [31]. A LED driver delivered excitation light at 465 nm and at 405 nm (~30–60 μW output at fiber tip). The light was reflected into a 200 µm 0.53 N.A. optic fiber patch-cord via Dual Fluorescence Minicube. Emissions were detected with a femtowatt photoreceiver (Model 2151, Newport). Sampling (12 kHz) and lock-in demodulation of the fluorescence signals were controlled by Doric Neuroscience Studio software with a decimation factor of 50. A behavioral camera synchronized the photometry recordings with time-locked behavioral tracking systems.

Fiber photometry data analysis was performed using custom-written Matlab scripts. The 405 nm channel was scaled to the 465 nm by applying a least mean squares linear regression. Scaled signals were used to calculate the ΔF/F_0_ where ΔF/F_0_ = (465 nm signal – fitted 405 nm signal)/fitted 405 nm signal. Z-scores were calculated using the average baseline of ΔF/F_0_ values from −1.0 s prior to onset of VLS (considered time zero, t = 0). The max and mean Z-scores were estimated between t = 0 and 10 s upon VLS and averaged/animal. The min Z-score for nest entry was estimated between t = 0 (nest entry) and 10 s upon nest entry and averaged per animal or −2 to 2 s to nest entry. The average Z-score was estimated pre VLS (−1 to 0 s), during VLS (+3 to +4s) and post VLS (+13 to +14 s).

### Optogenetics

Optic fiber implants were connected to a patch cable (Doric Lenses) and a commutator (rotary joint; LEDFRJ-B_FC for blue light and LEDFRJ-A_FC for yellow light, Doric Lenses), by means of an FC/SMC adapter to allow unrestricted movement [33]. Mice habituated for 8–10 min to the VLS apparatus without photostimulation. On day 1, mice freely explored the apparatus for 2–5 min before VLS were displayed. A high-power LED driver (DC2200, Thorlabs) was used to generate light pulses time-locked to VLS events at intensity ~2–5 mW at the fiber tip. Photoinhibition (593 nm, constant light) was delivered by an experimenter blind to animal conditions, 2 s prior, during and 2 s post each VLS event. On day 2, mice were subjected to the VLS paradigm with no light delivery. Day 3 followed the same light stimulation protocol as day 1. For optogenetic photostimulation without VLS, light pulses (473 nm, 20 Hz, 12 ms pulse, 3 s) were delivered at intervals >90 s on days 1 and 3. For optogenetic photostimulation paired with VLS, light pulses (473 nm, 20 Hz, 12 ms) were delivered 2 s prior, during and 2 s post each VLS event, on Days 2 and 3. All sessions were video recorded from above (HDR-CX4440 camera, SONY) and computationally analyzed with Ethovision XT.

### Immunostaining and microscopy

Immunohistochemistry and microscopy were performed as described previously [33]. Mice received sodium pentobarbital (200 mg/kg) and were transcardially perfused with ice-cold 0.1 M phosphate buffer saline (PBS, pH7.4) followed by 10 ml of cold 4% (W/V) paraformaldehyde (PFA). Brains were post-fixed in 4% PFA before transfering to 30% sucrose. Coronal sections (25 μm) were obtained using a freezing microtome (HM430; Thermo Fisher Scientific, MA, USA). Brain sections were permeabilized with 0.5% Triton X-100 (Sigma) for 10 min, blocked with 5% donkey serum (DS, Sigma) for 30 min and incubated with the primary antibody (rabbit anti-Sst, (1:700), RRID: sc-13099, anti-c-Fos, (1:500), Synaptic Systems, 226308) overnight at 4 °C. Slices were incubated in secondary antibody for 2 h (1:800; Life Technologies; donkey anti-rabbit 594, R37119, or goat anti-guinea pig 488, Invitrogen, AB_143165). Nuclei were counterstained with DAPI. Viral expression was visualized using the endogenous fluorescence of the virus. All slices were imaged using a fluorescent microscope (Zeiss, Carl Zeiss MicroImmagine, Inc., NY, USA) connected to computer-associated image analyzer software (Axiovision Rel., 4.6.1). For c-Fos quantification, images were processed in FIJI and manually counted.

### Statistical analysis

Data were analyzed using two-tailed unpaired *t*-tests, one- or two-way ANOVAs with/without repeated-measures (RM), or the restricted maximum likelihood (REML) mixed model, as indicated. Dunnett’s or Tukey’s *post hoc* tests were used for multiple comparisons. Two-tailed Pearson r was used for correlation analysis. Comparisons of Z-scores photometry signals were made using the calculated average for each animal. Sample sizes are estimated with α < 0.05 and β > 0.8 using G-power. Each data set was tested for normal distribution prior to analysis and presented as mean ± standard error of the mean (SEM). Principal component analysis was generated with a custom Python script using functions from the Scikit-learn and Matplotlib libraries. All statistical analyses and estimation of variation were performed in GraphPad Prism 10.1.0. Software (GraphPad Software Inc.) and statistical significance was established at *p* < 0.05.

## Results

### Innate adaptive defensive learning: mice adjust behavioral response to a potential threat

**Fig. 1:**
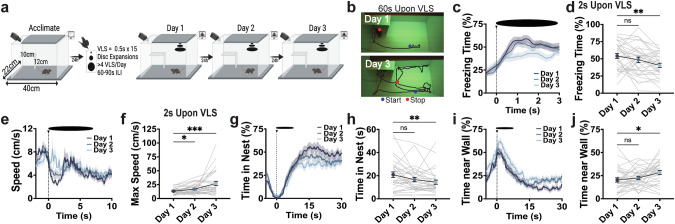
Mice exhibit innate defensive adaptive learning with repeated VLS exposures. **a** Schematic of VLS paradigm. **b** Representative example of day 1 and day 3 animals’ tracked behavior 60 s upon VLS. **c** Trace of freezing time (%) relative to VLS (t = 0), across 3 days. **d** Quantification of freezing time (%) 2 s upon VLS in **c**. One-way RM ANOVA (day effect: F_(2,92)_ = 7.082, *P* = 0.0025). Dunnett’s multiple comparisons ^**^p < 0.01. **e** Trace of speed (cm/s) relative to VLS (t = 0), across 3 days. **f** Quantification of max speed (cm/s) 2 s upon VLS in **e**. One-way RM ANOVA (day effect: F_(2,92)_ = 10.74, *P* = 0.0014). Dunnett’s multiple comparisons ^*^p < 0.05, ^***^p < 0.001. **g** Trace of time spent inside the nest (%) relative to VLS (t = 0), across 3 days. **h** Quantification of time in nest (s) after VLS across days. One-way RM ANOVA (day effect: F_(2,92)_ = 6.248, *P* = 0.0037). Dunnett’s multiple comparisons ^**^p < 0.01. **i** Trace of time spent near the wall (%) relative to VLS (t = 0), across 3 days. **j** Quantification of time spent near the wall (%), 30 s upon VLS in **i**. One-way RM ANOVA (day effect: F_(2,92)_ = 4.713, *P* = 0.0165). Dunnett’s multiple comparisons ^*^p < 0.05. (*n* = 31 mice). Data represent mean ± SEM.

To investigate an animal’s ability to adjust and optimize innate defensive behaviors, we implemented a multi-day VLS paradigm (Fig. 1a) and tracked the animal’s position throughout the VLS arena zones (Supplementary Fig. 1a and methods). The detection of a VLS from an overhead view triggered a sequence of defensive strategies that were significantly reduced if the same visual stimulus was presented from a side view (Supplementary Fig. 1b–f). Interestingly, we found that with multiple exposures of an overhead VLS for 3 consecutive days, mice learned to adjust VLS-evoked innate defensive behaviors (Fig. 1b–j). Immediate freezing significantly reduced (Fig. 1c, d), whereas speed increased across days (Fig. 1e, f). Mice continued to run with similar maximum speed (10 s upon VLS) and exhibited similar escape latencies to the nest across the 3 days (Supplementary Fig. 1g, h). Initially, animals spent a significant amount of time inside the nest, likely avoiding perceived threat, but gradually reduced their shelter time and began engaging in exploratory behaviors near the walls (Fig. 1g–j). We did not observe adaptive changes with time spent in the trigger zone, where VLS is presented (Supplementary Fig. 1i, j), or time in the safety zone (Supplementary Fig. 1k–l). Notably, adjustment of defensive responses was not detected within single-day trial sessions (Supplementary Fig. 2), suggesting that optimization of defensive strategies reflects learning and consolidation processes.

We performed linear correlation analysis to further investigate threat-evoked innate adaptive defensive learning (Supplementary Fig. 3a, b and Supplementary Tables 1–6). The latency to the nest showed strong negative correlation with the maximum speed the animals reached 10 s upon VLS, which was maintained across the 3 sessions, indicating the faster the animals ran, the earlier they entered the nest. Interestingly, immediate freezing predicted total time the animals would spend inside the nest. Time spent in the nest was also positively correlated with escape including latency to the nest and maximum speed 10 s upon VLS only on day 1. These results suggest that escape behaviors (i.e. running to the nest) and avoidance behaviors (i.e. time spent inside the nest) are related during initial sessions but become dissociable once animals learn to adjust defensive responses.

### Exposure to potential threat activates IPN GAD2 neurons and adjusts with defensive learning

**Fig. 2:**
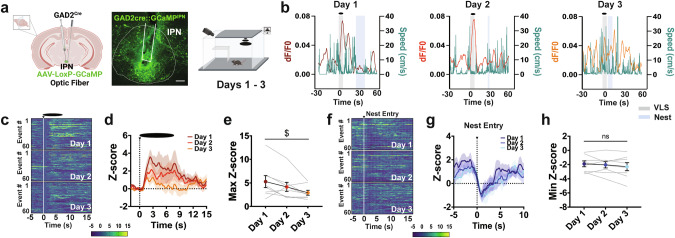
IPN GABAergic neurons respond to VLS and adapt with multiple exposures. **a** *Left*, schematic and representative image of GCaMP injection and fiber placement in the IPN in *GAD2 Cre* mice. Scale bar 100 μm. *Right*, schematic of VLS paradigm. **b** Example of speed trace (cm/s) compared to time-locked IPN *GAD2* fiber photometry signals (dF/F0) relative to VLS (t = 0), across 3 days. Heatmap representations **c** and Z-score values **d** of time-locked IPN *GAD2* neuronal activity relative to VLS (t = 0), across 3 days. **e** Quantification of responses in **d** as maximum Z-score values detected 10 s upon VLS. One-way RM ANOVA (day effect: F_(2,23)_ = 5.061, *P* = 0.0328). ^$^*p* < 0.05. Heatmap representations **f** and Z-score values **g** of time-locked IPN *GAD2* neuronal activity relative to the time of nest entry (t = 0), across 3 days. **h** Quantification of activity in **g** as minimum Z-score values detected 10 s upon nest entry. One-way RM ANOVA (day effect: F_(2,23)_ = 1.348, *P* = 0.2916). (*n* = 8 mice). Data represent mean ± SEM.

To study the neurocircuitry underlying the sequential innate defensive responses and adaptive threat learning, we focused on the IPN, an emerging region associated with anxiety and fear [34]. The IPN is an inhibitory nucleus highly enriched in GABAergic neurons that respond to aversive stimuli [35–37]. First, we utilized fiber photometry paired with VLS and expressed *Cre*-dependent GCaMP in the IPN of glutamic acid decarboxylase-2 Cre (*GAD2*^*Cre*^) mice to record IPN GABAergic activity during VLS events (Fig. 2a and Supplementary Fig. 4a). In response to overhead VLS, we detected a significant increase in IPN *GAD2* neuronal activity that was absent in control mice expressing *Cre*-dependent eGFP or if the same VLS was presented from a side view (Supplementary Fig. 4b, c). c-Fos expression analysis confirmed that VLS exposure increased activity in the IPN, as well as in other threat-associated regions such as the PAG and SC [6] (Supplementary Fig. 4d, e). Additional aversive stimuli, including a tail lift or foot shock, also increased IPN *GAD2* neuronal activation (Supplementary Fig. 4f, g). During the multi-day VLS paradigm we detected that IPN neuronal responses to VLS decreased across days as mice learned to optimize innate defensive strategies (Fig. 2b–e and Supplementary Fig. 4h). Other behaviors, such as rearing, also increased IPN activity, but these remained stable across the 3 days (Supplementary Fig. 4i), excluding the possibility of photobleaching. Reduced VLS-evoked IPN *GAD2* neuronal activation were not detected within a trial session (Supplementary Fig. 4j, k).

Interestingly, IPN *GAD2* neuronal activity dynamics inversely mirrored changes in speed during VLS events (Fig. 2b), as well as across the whole VLS session (Supplementary Fig. 5a). Correlation analysis demonstrated VLS-induced activation levels of IPN *GAD2* neurons negatively related with speed 10 s post-VLS initiation (Supplementary Fig. 5b). Furthermore, VLS-induced engagement of IPN *GAD2* neurons positively correlated with latency to nest (Supplementary Fig. 5c) and time spent near walls (Supplementary Fig. 5d). Notably, we also detected reduced activity of IPN GABAergic neurons when mice entered the nest (Fig. 2f–h), although these signals did not adjust across sessions. Altogether these results suggested that IPN *GAD2* neuronal activity reflected VLS-evoked defensive actions.

### Silencing IPN GAD2 neurons during VLS presentations reduces innate defensive behaviors

**Fig. 3:**
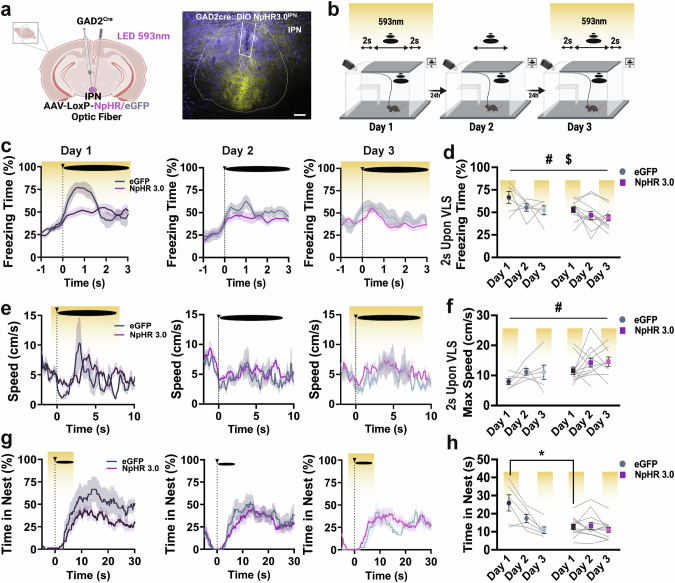
Photoinhibition of IPN GABAergic neurons reduces defensive behaviors. **a** Schematic and representative image of NpHR3.0 injection and fiber placement in the IPN in *GAD2 Cre* mice. Scale bar 100 μm. **b** Schematic representation of 3-day VLS paradigm with NpHR inhibition on days 1 and 3 (yellow). **c** Traces of freezing time (%) relative to VLS (t = 0), across 3 days in IPN *GAD2* eGFP and NpHR animals. **d** Quantification of freezing time (%) 2 s upon VLS in **c**. Two-way RM ANOVA (day effect: F_(2,32)_ = 3.928, *P* = 0.0312; treatment effect: F_(1,16)_ = 6.679, *P* = 0.0200; interaction: F_(2,32)_ = 0.2304, *P* = 0.7956), treatment effect ^#^*p* < 0.05, day effect ^$^*p* < 0.05. **e** Traces of speed (cm/s) relative to VLS (t = 0), across 3 days in IPN *GAD2* eGFP and NpHR animals. **f** Quantification of max speed (cm/s) 2 s upon VLS in **e**. Two-way RM ANOVA (day effect: F_(2,32)_ = 2.299, *P* = 0.1185; treatment effect: F_(1,16)_ = 6.514, *P* = 0.0213; interaction: F_(2,32)_ = 0.0294, *P* = 0.9711), treatment effect ^#^*p* < 0.05. **g** Trace of time spent inside the nest (%) relative to VLS (t = 0), across 3 days in IPN *GAD2* eGFP and NpHR animals. **h** Quantification of time in nest (s) after VLS across days. Two-way RM ANOVA (day effect: F_(2,32)_ = 17.02, *P* < 0.0001; treatment effect: F_(1,16)_ = 4.336, *P* = 0.0537; interaction: F_(2, 32)_ = 11.45, *P* = 0.0002), Tukey’s multiple comparisons ^*^p < 0.05. (*n* = 6 eGFP and 12 NpHR3.0 mice). Data represent mean ± SEM.

To determine the functional implication of IPN *GAD2* neurons in defensive responses, we selectively silenced these neurons during VLS presentations. *GAD2*^*Cre*^ mice were injected with *Cre*-dependent halorhodopsin (NpHR) or eGFP and implanted with an optic fiber in the IPN (Fig. 3a and Supplementary Fig. 6a). Animals then underwent the 3-day VLS paradigm with photoinhibition 2 s prior to VLS, which remained ON until 2 s post-VLS, on days 1 and 3 (Fig. 3b). If activation of IPN GABAergic neurons is important for the expression of innate defensive responses, then silencing these neurons on Day 1, when this circuit is highly engaged by VLS, should cause a decrease in defensive behaviors. In contrast, photoinhibition on Day 3 would inform if these neurons are involved in recalling a learned innate threat memory. Compared to controls, IPN NpHR animals exhibited a decrease in VLS-induced immediate freezing response (Fig. 3c, d) along with increased speed upon VLS display (Fig. 3e, f), but intact nest latency (Supplementary Fig. 6b). Additionally, silencing IPN *GAD2* neurons led to decreased time spent in the nest on day 1 of the looming session (Fig. 3g, h); instead, animals remained in the vicinity of the safety zone (Supplementary Fig. 6c, d). Other behavioral responses, including time spent near walls (Supplementary Fig. 6e, f) or in the trigger area (Supplementary Fig. 6g, h), were not significantly affected by IPN *GAD2* photoinhibition. Applying principal component analysis (PCA) to the multidimensional behavioral dataset across VLS sessions further demonstrated that different behavioral variables cluster differently between control and NpHR groups (Supplementary Fig. 7).

We also tested if activating IPN GABAergic neurons triggered innate defensive strategies. To this aim, we injected the IPN of *GAD2*^*Cr*e^ mice with *Cre*-dependent channelrhodopsin (ChR2) and implanted an optic fiber in the target site (Supplementary Fig. 8a). First, we tested whether photostimulation of IPN *GAD2* neurons without VLS was sufficient to elicit defensive behaviors (Supplementary Fig. 8b, c). Optogenetic excitation alone did not produce differences between groups for the defensive responses measured (Supplementary Fig. 8d–f). Next, we investigated whether optogenetically maintaining IPN GABAergic activity during VLS presentations on Days 2–3 would prevent behavioral adaptation. Mice receiving IPN photostimulation exhibited reduced latencies to enter the nest and a trend toward spending more time spent inside the nest across VLS days (Supplementary Fig. 8g–k), demonstrating impaired threat adaptation.

### IPN→LDTg GAD2 neurons are engaged by VLS and reduce activation with innate defensive adaptive learning

**Fig. 4:**
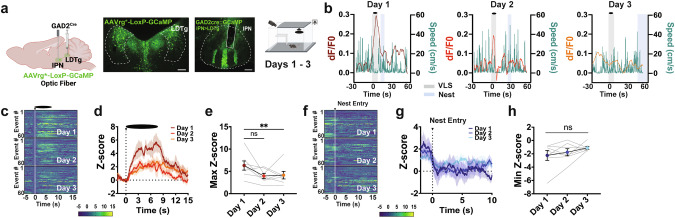
IPN GAD2 neurons projecting to the LDTg are engaged by VLS and adapt with multiple exposures. **a** *Left*, schematic of retrograde viral injection and optic fiber strategy used. *Middle*, representative images of retroviral mediated GCaMP injection in the LDTg and fiber placement in the IPN in *GAD2 Cre* mice. Scale bar 100 μm. *Right*, schematic of VLS paradigm. **b** Example of speed trace (cm/s) compared to time-locked IPN→LDTg *GAD2* fiber photometry signals (dF/F0) relative to VLS (t = 0), across 3 days. Heatmap representations **c** and Z-score values **d** of time-locked IPN→LDTg *GAD2* neuronal activity relative to VLS (t = 0), across 3 days. **e** Quantification of responses in **d** as maximum Z-score values detected 10 s upon VLS. One-way RM ANOVA (day effect: F_(2,20)_ = 5.409, *P* = 0.050), Dunnett’s multiple comparisons. ^**^p < 0.01. Heatmap representations **f** and Z-score values **g** of time-locked IPN→LDTg *GAD2* neuronal activity relative to the time of nest entry (t = 0), across 3 days. **h** Quantification of activity in **g** as minimum Z-score values detected 10 s upon nest entry. One-way RM ANOVA (day effect: F_(2,20)_ = 1.856, *P* = 0.2059). (*n* = 7 mice). Data represent mean ± SEM.

The IPN projects to brain regions associated with fear and anxiety including the LDTg [29, 30]. To test if the IPN innervates the LDTg to convey innate defensive behaviors, we recorded activity dynamics in the IPN of *GAD2*^*Cre*^ mice bilaterally injected the LDTg with a retrogradely-transported AAVrg *Cre*-dependent GCaMP (Fig. 4a and Supplementary Fig. 9a). An overhead VLS elevated activity of *GAD2*^IPN→LDTg^ neurons that significantly decreased over consecutive sessions (Fig. 4b–e and Supplementary Fig. 9b). No reductions were detected within sessions (Supplementary Fig. 9c, d). *GAD2*^IPN→LDTg^ circuit activity inversely mirrored changes in speed (Fig. 4b) and negatively correlated with speed levels (Supplementary Fig. 9e) but exhibited no correlation with latency to nest (Supplementary Fig. 9f), although *GAD2*^IPN→LDTg^ activation positively predicted time spent near the walls (Supplementary Fig. 9g). Similar to IPN *GAD2* measures, *GAD2*^IPN→LDTg^ neurons showed a decrease in circuit activity when animals entered the nest which was sustained across sessions (Fig. 4f–h), overall indicating that dynamics of *GAD2*^IPN→LDTg^ activation encodes threat processing.

### Silencing IPN GAD2 neuron→LDTg projections impairs innate defensive adaptive learning

**Fig. 5:**
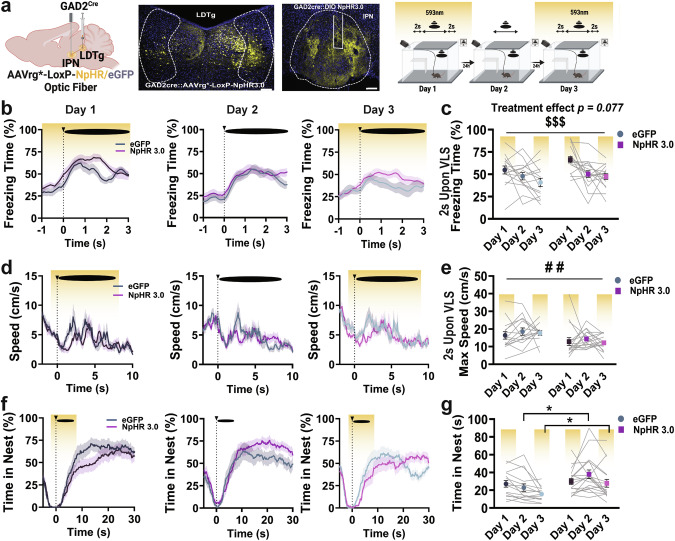
IPN→LDTg GAD2 neuronal circuit controls defensive adaptive learning. **a** *Left*, schematic and representative image of retrograde NpHR3.0 injection in the LDTg and fiber placement in the IPN in *GAD2 Cre* mice. Scale bar 100 μm. *Right*, schematic representation of 3-day VLS paradigm with NpHR inhibition on days 1 and 3 (yellow). **b** Traces of freezing time (%) relative to VLS (t = 0), across 3 days in IPN→LDTg *GAD2* eGFP and NpHR animals. **c** Quantification of freezing time (%) 2 s upon VLS in **b**. Two-way RM ANOVA (day effect: F_(2,60)_ = 11.16, *P* = 0.0001; treatment effect: F_(1,30)_ = 3.338, *P* = 0.0777; interaction: F_(2,60)_ = 0.7984, *P* = 0.4548), day effect ^$$$^*p* < 0.001. **d** Traces of speed (cm/s) relative to VLS (t = 0), across 3 days in IPN→LDTg *GAD2* eGFP and NpHR animals. **e** Quantification of max speed (cm/s) 2 s upon VLS in **d**. Two-way RM ANOVA (day effect: F_(2,60)_ = 0.6815, *P* = 0.4984; treatment effect: F_(1,30)_ = 9.599, *P* = 0.0042; interaction: F_(2,60)_ = 0.1906, *P* = 0.8270), treatment effect ^##^*p* < 0.01. **f** Trace of time spent inside the nest (%) relative to VLS (t = 0), across 3 days in IPN→LDTg *GAD2* eGFP and NpHR animals. **g** Quantification of time in nest (s) after VLS across days. Two-way RM ANOVA (day effect: F_(2,60)_ = 6.927, *P* = 0.0020; treatment effect: F_(1,30)_ = 3.496, *P* = 0.0713; interaction: F_(2,60)_ = 3.192, *P* = 0.0481), Tukey’s multiple comparisons ^*^p < 0.05. (*n* = 15 eGFP and 17 NpHR3.0 mice). Data represent mean ± SEM.

To test if IPN GABAergic neurons projecting to the LDTg are necessary for innate adaptive defensive responses, we used time-locked optogenetic approaches in a circuit-specific manner. We injected *Cre*-dependent AAVrg NpHR or eGFP into the LDTg and placed an optic fiber in the IPN (Fig. 5a and Supplementary Fig. 10a). Experimental and control mice demonstrated a significant reduction in freezing behavior across days (Fig. 5b, c). However, compared to controls, optogenetic silencing of the IPN→LDTg circuit slightly increased freezing behavior (Fig. 5b, c) and significantly reduced max speed 2 s upon VLS events (Fig. 5d, e). Noticeably, control mice significantly reduced time spent in the nest after VLS presentation across sessions, whereas mice with *GAD2*^IPN→LDTg^ circuit photoinhibition continued spending a significant amount of time inside the nest over VLS days (Fig. 5f, g), suggesting this circuit is involved in adaptive threat learning. Time spent in other zones were not influenced by optogenetic inhibition of the *GAD2*^IPN→LDTg^ circuit (Supplementary Fig. 10b–h).

### Sst IPN neurons encode defensive responses in a threatening environment

**Fig. 6:**
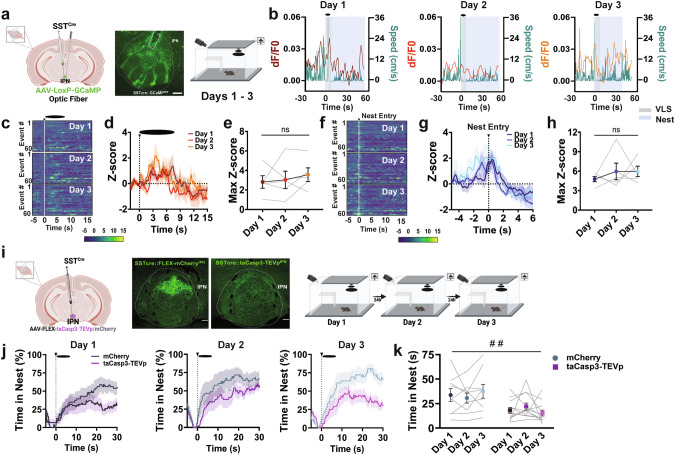
IPN Sst+ neurons encode threat and avoidance behaviors. **a** *Left*, schematic of viral injection and fiber placement; representative image of viral-mediated GCaMP expression and fiber placement in IPN of Sst^Cre^ mice. Scale bar 100 μm. *Right*, VLS paradigm. **b** Representative traces of IPN Sst^+^ activity recordings (dF/F0) compared to speed (cm/s) relative to VLS presentations (t = 0), across days 1–3. Heatmap representations **c** and Z-score values **d** of time-locked IPN Sst^+^ neuronal activity relative to VLS (t = 0), across 3 days. **e** Quantification of responses in **d** as maximum Z-score values detected 10 s upon VLS. One-way RM ANOVA (day effect: F_(2,14)_ = 0.4439, *P* = 0.6262). Heatmap representations **f** and Z-score values **g** of time-locked IPN Sst^+^ neuronal activity relative to the time of nest entry (t = 0), across 3 days. **h** Quantification of activity in **g** as maximum Z-score values detected during nest entry. One-way RM ANOVA (day effect: F_(2,14)_ = 0.5364, *P* = 0.5166). (*n* = 5 mice). **i** *Left*, Schematic of taCasp3-TEVp injection in the IPN in *Sst Cre* mice and representative image of Sst immunostaining (green) in the IPN of control and ablation animals. Scale bar 50 μm. *Right*, Schematic representation of 3-day VLS paradigm. **j** Trace of time spent inside the nest (%) relative to VLS (t = 0), across 3 days in IPN Sst mCherry and taCasp3-TEVp animals. **k** Quantification of time in nest (s) after VLS across days. Two-way RM ANOVA (day effect: F_(2,38)_ = 0.01528, *P* = 0.9846; treatment effect: F_(1,19)_ = 8.379, *P* = 0.0093; interaction: F_(2, 38)_ = 2.141, *P* = 0.1315), treatment effect ^##^*p* < 0.01. (*n* = 9 mCherry and 12 taCasp3-TEVp mice). Data represent mean ± SEM.

Within the IPN, a GABAergic subpopulation expressing somatostatin (Sst) [37, 38] demonstrates a highly selective dorso-rostral to ventro-caudal gradient (Supplementary Fig. 11a). Sst is a neuropeptide typically co-released with GABA and involved in anxiety-like behaviors [39]. Interestingly, tracing analysis demonstrated that IPN Sst+ neurons are projecting neurons innervating the LDTg (Supplementary Fig. 11b, c). To explore whether IPN Sst^+^ responded to VLS, we injected Cre-dependent GCaMP in the IPN of mice expressing Cre under the control of the Sst promoter (Fig. 6a and Supplementary Fig. 11d, e). Using this approach, we previously demonstrated that ~60% of Sst neurons express the GCaMP biosensor across the rostral to caudal IPN [37]. We found that aversive stimuli such as VLS presentations (Fig. 6b–e and Supplementary Fig. 11f), a tail lift (Supplementary Fig. 11g) or foot shock (Supplementary Fig. 11h), triggered increases in IPN Sst^+^ neuronal activity. Remarkably, VLS-induced IPN Sst^+^ activation occurred after reaching max speed upon VLS (Fig. 6b) and did not reduce from day 1 to day 3 (Fig. 6c–e). Interestingly, we also detected IPN Sst^+^ activation time-locked to nest entry, although these signals did not adjust across repeated VLS sessions (Fig. 6f–h). Further analysis demonstrated that on day 1, the engagement of IPN Sst^+^ neurons with nest entry positively correlated with speed levels 10 s upon VLS (Supplementary Fig. 11i) and time spent inside the nest (Supplementary Fig. 11j), while it negatively correlated with nest latency (Supplementary Fig. 11k) or time spent near the walls (Supplementary Fig. 11l), suggesting these neurons may encode avoidance aspects of threat processing.

### Genetic ablation of IPN Sst+ neurons reduces avoidance from perceived threat

To test the role of IPN Sst^+^ neurons in threat processing and defensive learning, we genetically ablated them using *Cre*-dependent caspase 3 (Fig. 6i and Supplementary Fig. 12a, b). IPN Sst^+^ neuron ablation did not affect VLS-induced freezing (Supplementary Fig. 12c, d) or changes in speed (Supplementary Fig. 12e, f) across the 3-day looming sessions. Nevertheless, we observed that IPN Sst^+^ ablated animals significantly reduced time spent inside the nest as compared to mCherry controls (Fig. 6j, k). In contrast, animals with IPN Sst ablation spent more time nearby safety (Supplementary Fig. 12g, h) and wall zones (Supplementary Fig. 12i, j). Reduced nest time in mice with ablated IPN Sst+ neurons suggest these neurons contribute to processing threat avoidance, but not motor function. Indeed, we also detected that IPN Sst^+^ ablation increased time spent in open arms of the elevated plus maze (Supplementary Fig. 12k) but not closed arm entries (Supplementary Fig. 12l). Similarly, IPN Sst ablated animals showed increased center exploration time in an open field test (Supplementary Fig. 12m) without altering locomotion (Supplementary Fig. 12n).

## Discussion

Defensive behaviors are regulated by adaptive mechanisms contingent on the previous experience of an aversive outcome. Here we define sequences of threat stimulus-evoked responses that adjust to repeated VLS exposures when there is no evidence of real harm. We identify the IPN as a critical node orchestrating adaptive defensive strategies. Inhibitory projections from the IPN to the LDTg control the learning aspect of threat processing. In contrast, a subpopulation of IPN neurons expressing Sst mediate generalized aspects of avoidance-related behaviors. Our findings help elucidate underlying neurobiology of innate fear behaviors and how they are regulated in the absence of danger.

The selection of ongoing defensive behaviors is constantly updated by recent experiences that contribute to perceptual and value-based decision-making and action selection [40, 41]. We found that upon repeated VLS sessions, mice reduced immediate freezing, as well as avoidance behaviors including time spent inside shelter while switching behavioral strategies to engage in more exploratory actions. Importantly, changes in defensive strategies were detected across but not within daily sessions suggesting shifting strategies may reflect learning and consolidation processes [40]. Although most adaptive defensive behaviors to VLS have focused on escape responses [40, 42], emotional avoidance components of threat processing remain understudied. Our behavioral analysis revealed a dissociation between time spent inside shelter and escape responses over repeated VLS sessions. Escape is a flexible behavior under cognitive control [41]. Integrating variables of escape behavior together with avoidance and the engagement of exploratory strategies upon threat assessment -for instance when animals leave shelter to take new risks-, is necessary to understand how these are coordinated to drive adaptive behaviors. Given that the speed of habituation to VLS depends on the behavioral context [43] and is stimulus-specific [40], future studies should help elucidate sequence actions of threat adaptation specific to the context or type of threatening stimuli.

Emerging evidence indicates that divergent circuits orchestrate escape and freezing responses to VLS [2, 7, 44, 45]. Our data indicate that IPN GABAergic neurons are recruited by innate visual threat and exhibit neuronal adaptation with defensive learning. These results support that the habenular axis is involved in anxiety [16–23] and conditioned fear-related behaviors [24–27]. Moreover, they demonstrate that activity of IPN GABAergic neurons adapt over repeated sessions with cues of both negative and positive values, as we have shown for rewarding novel social interactions [31], supporting a role of the IPN in familiarity signaling [33]. IPN excitability is tightly regulated by cholinergic and glutamatergic input from the mHb [24, 46]. These presynaptic mHb terminals, in turn, are modulated by retrograde GABA release from IPN neurons acting on presynaptic GABA_B_ receptors [25], as well as by Sst and nitric oxide release from the IPN, which act retrogradely on mHb presynaptic terminals to control excitatory neurotransmission [47]. The Ca^2+^-permeable AMPA receptor-dependent release of GABA from IPN neurons and retrograde GABA_B_ activation on mHb terminals results in a long-lasting enhancement of glutamate release, which is essential for conditioned fear extinction [48]. Future studies should test if this plasticity also underlies innate threat adaptation to VLS.

Silencing overall IPN GABAergic activity during VLS presentations devalued the stress component of a VLS threat and reduced freezing responses and avoidance behaviors. Conversely, photoactivation of IPN GABAergic neurons on days 2 and 3 of the VLS prevented threat adaptation, similarly to optogenetic effects on conditioned learned fear [28]. As opposed to ventral tegmental area (VTA) GABAergic neurons [49], brief optogenetic stimulation of IPN GABAergic subpopulation was insufficient to trigger early or late defensive behaviors, indicating specialized roles of VTA and IPN inhibitory networks. Beyond simply integrating negative stimuli, our results support the IPN as an aversion amplifier [28], which has important implications for affective responses. Considering that the habenula-interpeduncular circuit also modulates innate fear behaviors triggered by predator odor [50], our findings reinforce the notion that IPN circuits may be recruited by a variety of natural threats across multiple sensory modalities, integrating both visual and olfactory cues.

Here, we report that activity dynamics of IPN GABAergic neurons inversely mirrors changes in speed. The IPN is part of the NI network that controls locomotor speed, arousal, and hippocampal theta rhythms [51]. We previously showed that activity of IPN GABAergic neurons is reduced during novel social investigations [31], sucrose consumption and grooming events [37], behavioral episodes where there is an absence of locomotion. Here, we found reduced IPN GABAergic neuronal activity with nest entries -when animals lower speed-, suggesting a role of the IPN beyond controlling locomotion to also processing affective behaviors like avoidance, approach or consummatory events. Freezing is cardinal in stress-coping processes as it corresponds to a state of hypervigilance that enables decision-making [2]. Spending time inside the nest upon VLS could also represent a stress-coping mechanism that prepares for future exploratory actions. Altogether, our results support the view that the IPN responds to aversive stimuli to regulate distinctive stress-related coping strategies [37]. One limitation of the current study is that most experiments were conducted using male mice. Previous research has reported sex differences in IPN neuronal excitability and anxiety-like behaviors [52, 53]. Additionally, the isolating conditions of animal housing can enhance threat responses [40]. Future investigations are needed to determine sex-specific effects of threat adaptation and whether housing conditions influence IPN activation levels.

IPN GABAergic neurons send strong inhibitory projections to the raphe, tegmentum and NI [29, 30]. Through the LDTg, the IPN controls nicotine aversion [32] or social novelty preference [31]. We found IPN→LDTg circuit engagement with aversive VLS and neuronal adaptation with multiple exposures. However, optogenetic silencing the IPN→LDTg circuit increased freezing and impaired defensive adaptive learning specifically for time spent in shelter. These responses differed from those observed when manipulating overall IPN GAD2 neuronal populations, suggesting that the IPN may rely on other projections such as to the NI, which is implicated in amplifying fear responses [28] and could contribute to controlling expression of innate VLS threat responding. LDTg GABAergic neurons inhibit VTA to promote unconditioned freezing responses [54]. Although work from our group and others suggest IPN GABAergic neurons inhibit LDTg cholinergic neurons innervating the VTA [31, 32], we cannot exclude the possibility the IPN inhibits other LDTg neurons to regulate fear and freezing responses. Considering different LDTg interneurons oppositely regulate innate fear [55], understanding how the IPN controls LDTg function and network connectivity is necessary to elucidate the critical role of this circuit in innate defensive adaptive learning.

Among all IPN GABAergic cell types, we recently demonstrated Sst IPN neurons are activated by acute stress to drive motivational behaviors [37]. Our fiber photometry data revealed activation of IPN Sst neurons during threatening VLS, (although milder compared to overall IPN GAD2 neurons), but also activation when animals entered the shelter to engage avoidance behaviors. Thus, it is possible that different IPN Sst populations exist to control various aspects of threat responding, avoidance and coping mechanisms. Indeed, genetic ablation of IPN Sst neurons reduced time inside shelter while also affecting general anxiety-like behaviors. Sst is highly enriched in the alpha5-^Amigo1^ IPN cell population that projects to midbrain and hindbrain areas [47]. Future studies should explore potentially differential roles of these projections in threat processing and learning.

Collectively, our findings implicate the IPN as a critical node of innate threat-processing and adaptive defensive learning, suggesting IPN dysregulation may contribute to numerous psychiatric disorders associated with threat maladaptation such as post-traumatic stress disorder.

## Supplementary information

Supplementary Information

## Data Availability

All data will be available upon request to the corresponding author.

## Code availability

The codes used to process and analyze fiber photometry and Ethovision data will be made available on https://github.com/MolasLAB.
