# Supplementary material for: Interpeduncular GABAergic neuron function controls threat processing and innate defensive adaptive learning
Source: Mol Psychiatry. 2025 Aug 8;30(11):5427–36. doi: 10.1038/s41380-025-03131-9 (PMC12532598; doi:10.1038/s41380-025-03131-9)
Supplement: Supplementary file 1 — Supplementary Information [file 41380_2025_3131_MOESM1_ESM.docx]

**Interpeduncular GABAergic neuron function controls threat processing and innate defensive adaptive learning**

Elora W Williams, B.S.^1,2^ ; Leshia Snively, B.A.^1^ ; Benjamin R O’Meara, B.A.^1,2^ ; Hannah L Jacobs, B.A.^1,2^ ; Miranda Kolb, B.A.^1,2^ ; Rubing Zhao-Shea, Ph.D.^3^ ; Rebecca G Pavchinskiy, B.S.^3^; Emma Keppler B.S.^1,2^; Michael V Baratta, Ph.D.^2^ ; Andrew R Tapper, Ph.D.^3^ and Susanna Molas, Ph.D.^1,2,3,4^

^1^Institute for Behavioral Genetics, University of Colorado Boulder, 1480 30^th^ St, Boulder, 80303, CO, USA.

^2^Department of Psychology and Neuroscience, University of Colorado Boulder, 1905 Colorado Ave, Boulder, 80309, CO, USA.

^3^Department of Neurobiology, Brudnick Neuropsychiatric Research Institute, University of Massachusetts Chan Medical School, 364 Plantation St, LRB, Worcester, 01605, MA

^4^Crnic Institute Boulder Branch, BioFrontiers Institute, University of Colorado Boulder, 3415 Colorado Avenue, Boulder, 80303, CO, USA.

**Corresponding author:**

**Susanna Molas, PhD**

**Institute for Behavioral Genetics/Department of Psychology and Neuroscience, University of Colorado Boulder, Boulder, CO**

**Email:**

[**susanna.molas@colorado.edu**](mailto:susanna.molas@colorado.edu)

# SUPPLEMENTARY FIGURES

## Suppl. Figure 1. Visual threat perception and adaptive learning.

**|** (**a**) Representative image of VLS apparatus with defined zones. (**b**) Schematic of the overhead (top) and side (bottom) VLS paradigms. (**c**) Freezing time (%) 2 sec upon VLS, unpaired t-test (t_(37)_ = 2.046, *P* = 0.0479), (**d**) max speed (cm/s) 10 sec upon VLS, unpaired t-test (t_(37)_ = 2.557, *P* = 0.0148), (**e**) latency to nest (s) (t_(37)_ = 1.755, *P* = 0.0875) and (**f**) time spent inside the nest (s), unpaired t-test (t_(37)_ = 3.224, *P* = 0.0026), between overhead and side VLS groups. * p < 0.05, ** p < 0.01. (**g**) Quantification of max speed (cm/s) 10s upon VLS across 3 days in (figure 1e). One-way RM ANOVA (day effect: F_(2,92)_ = 1.680, *P* = 0.2038). (**h**) Latency to nest (s) relative to VLS, across 3 days. One-way RM ANOVA (day effect: F_(2,92)_ = 0.023, *P* = 0.9647). (**i**) Trace of time spent in the trigger zone (%) relative to VLS, across 3 days. (**j**) Quantification of time spent in the trigger area (%) 30 sec upon VLS in (i). One-way RM ANOVA (day effect: F_(2,92)_ = 0.061, *P* = 0.8902). (**k**) Trace of time spent in the safe zone (%) relative to VLS, across 3 days. (**l**) Quantification of time spent in the safe zone 30 sec upon VLS in (k). One-way RM ANOVA (day effect: F_(2,92)_ = 0.488, *P* = 0.5924). (n = 31 mice). Data represent mean ± SEM.

*
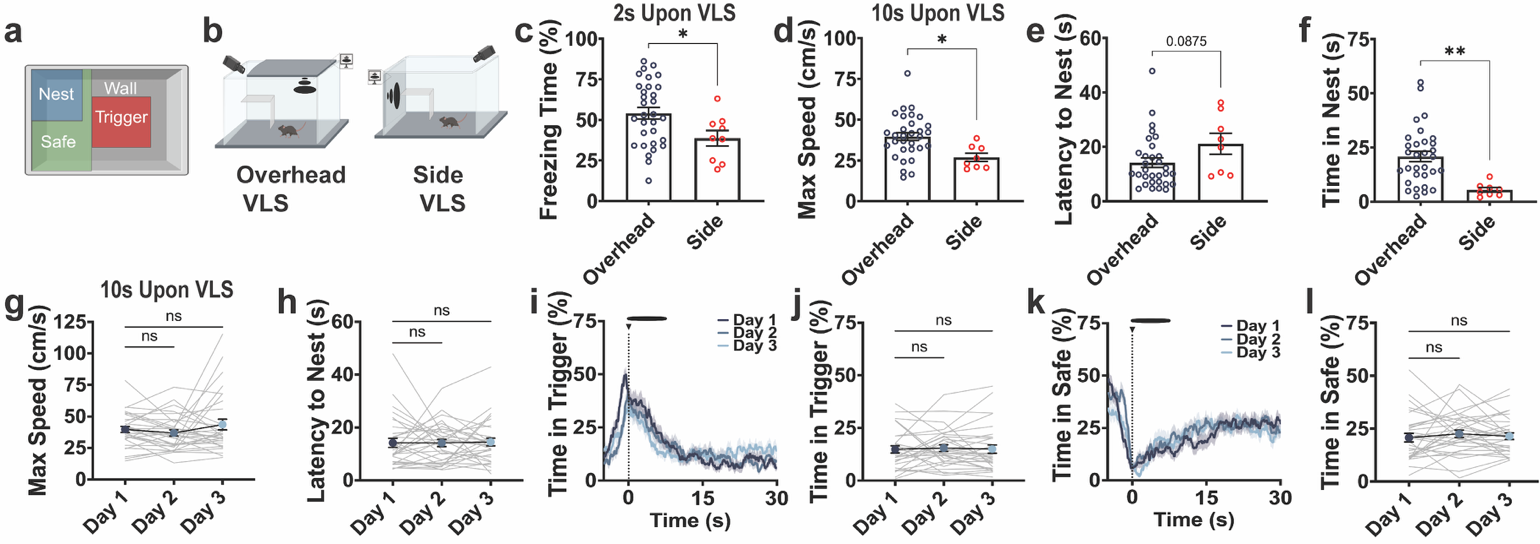
*

## Suppl. Figure 2. Defensive adaptive learning does not occur within a trial session.

**|** (**a**) Freezing time (%) 2s upon VLS across events for day 1 (REML, time effect: F_(5.088, 132.3)_ = 0.8472, *P* = 0.5203), day 2 (REML, time effect: F_(4.547, 119.0)_ = 1.156, *P* = 0.3347) and day 3 (REML, time effect: F_(5.015, 123.7)_ = 2.070, *P* = 0.0734). (**b**) Max speed (cm/s) 2s upon VLS across events for day 1 (REML, time effect: F_(4.871, 126.7)_ = 0.335, *P* = 0.8868), day 2 (REML, time effect: F_(3.855, 100.9)_ = 0.8318, *P* = 0.5044) and day 3 (REML, time effect: F_(3.009, 74.22)_ = 0.4555, *P* = 0.7148). (**c**) Max speed (cm/s) 10s upon VLS across events for day 1 (REML, time effect: F_(2.915, 75.80)_ = 1.490, *P* = 0.2248), day 2 (REML, time effect: F_(4.359, 114.1)_ = 0.3308, *P* = 0.8715) and day 3 (REML, time effect: F_(3.291, 81.18)_ = 0.4586, *P* = 0.7295). (**d**) Latency to nest (s) across events for day 1 (REML, time effect: F_(3.436, 87.63)_ = 1.147, *P* = 0.3376), day 2 (REML, time effect: F_(3.689, 97.76)_ = 0.4151, *P* = 0.7823) and day 3 (REML, time effect: F_(4.189, 104.7)_ = 1.258, *P* = 0.2906). (**e**) Time in nest (s) across events for day 1 (REML, time effect: F_(3.297, 86.27)_ = 2.513, *P* = 0.0584), day 2 (REML, time effect: F_(4.199, 113.4)_ = 0.8134, *P* = 0.5242) and day 3 (REML, time effect: F_(3.426, 90.21)_ = 0.9577, *P* = 0.425). (**f**) Time near the wall (%) across events for day 1 (REML, time effect: F_(4.002, 104.1)_ = 1.321, *P* = 0.2669), day 2 (REML, time effect: F_(4.135, 108.2)_ = 0.6265, *P* = 0.6499) and day 3 (REML, time effect: F_(4.111, 101.4)_ = 1.172, *P* = 0.328). (n = 31 mice). Data represent mean ± SEM.

*
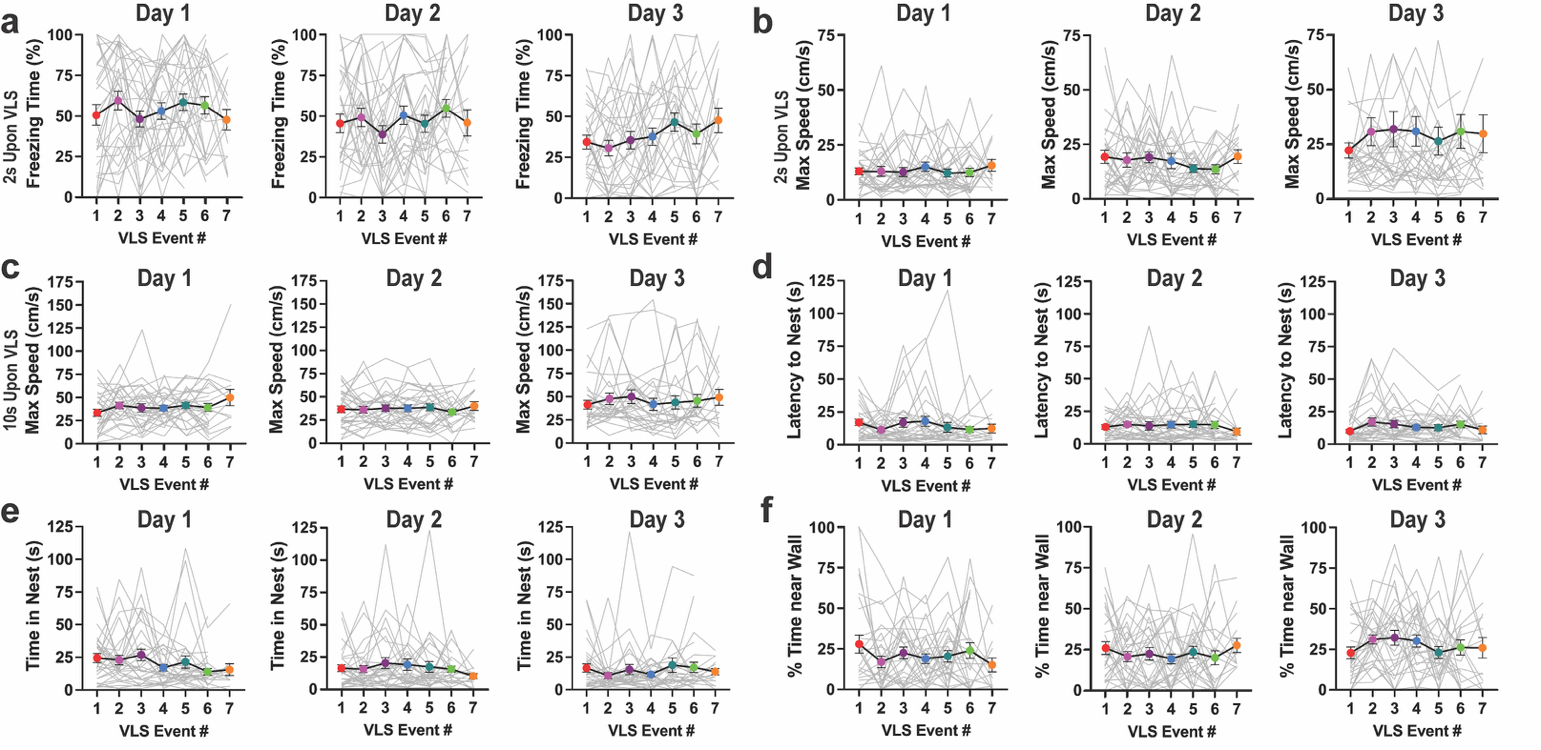
*

## Suppl. Figure 3. Predictive variables of defensive responses and threat adaptation.

**|** Correlation heatmaps reporting Pearson’s correlation coefficients (**a**) and *p* values (**b**) for each comparison for days 1-3. *p < 0.05. (n = 189 – 192 VLS events/day, from n = 31 mice)

*
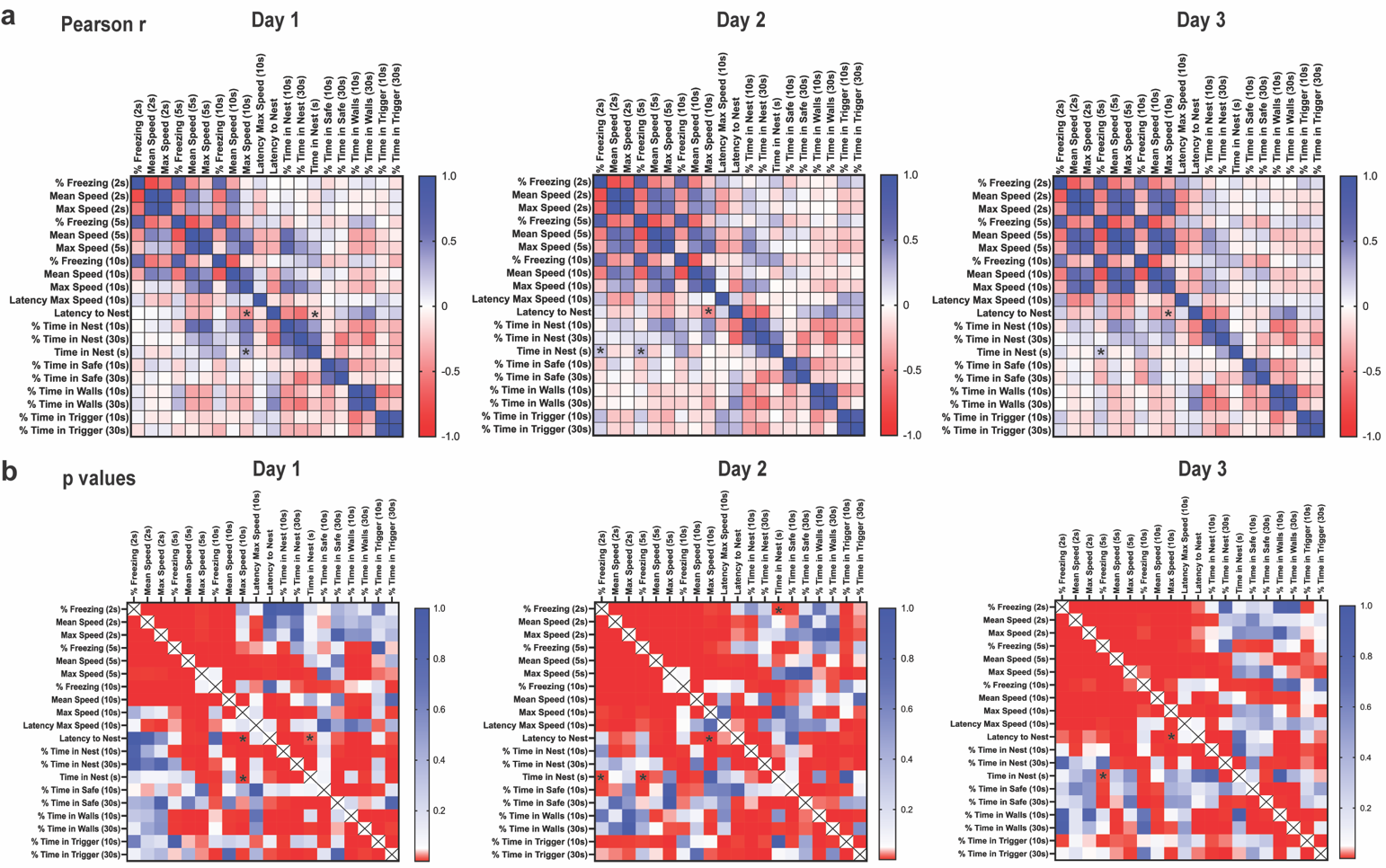
*

## Suppl. Figure 4. Aversive stimuli increase activity of IPN GAD2 neurons.

**|** (**a**) Representative images and schematics of viral injections and fiber implants, scale bars 100µm. (**b**) *Left*, schematics of the overhead and side VLS paradigms. *Right*, Z-score values of time-locked IPN *GAD2* neuronal activity relative to VLS (t = 0) presented from an overhead or side view. (**c**) Z-score values of IPN *GAD2* neuronal activity relative to VLS (t = 0) in mice expressing GCaMP or eGFP. (**d**) *Left*, representative images of c-Fos expression in the IPN of control (CT) and VLS groups, scale bars 100µm. *Right*, quantification of c-Fos neurons per IPN area, unpaired t-test (t_(5)_ = 3.442, *P* = 0.0184) (n= 3-4 mice/group). (**e**) Representative images of c-Fos expression in the periaqueductal gray (PAG) (*left*) and the superior colliculus (SC)(*right*) of CT and VLS groups, scale bars 100µm. IPN *GAD2* neuronal activity responses upon a tail lift (**f**) or a foot shock (**g**). (**h**) Mean Z-score values of IPN GAD2 activity pre (-1 to 0 sec), during (+3 to +4 sec) and post (+13 to +14 sec) VLS events, across day 1 (RM ANOVA, time effect: F_(1.232, 8.623)_ = 5.443, *P* = 0.0407), day 2 (RM ANOVA, time effect: F_(1.710, 11.97)_ = 2.636, *P* = 0.1177) and day 3 (RM ANOVA, time effect: F_(1.703, 11.92)_ = 1.899, *P* = 0.1943)(n = 8 mice). (**i**) IPN *GAD2* neuronal activity responses upon rearing behavior across 3 days. (**j**) Z-score traces of IPN *GAD2* neuronal activity across VLS events within a session, for 3 consecutive days. (**k**) Maximum Z-score values of IPN *GAD2* neuronal activity levels across VLS events on day 1 (REML, time effect: F_(2.236, 15.28)_ = 1.262, *P* = 0.3148), day 2 (REML, time effect: F_(3.294, 22.51)_ = 1.631, *P* = 0.2076) and day 3 (REML, time effect: F_(3.067, 18.40)_ = 0.951, *P* = 0.4380). (n = 4 – 7 VLS events/day, from n = 8 mice). Data represent mean ± SEM.

*
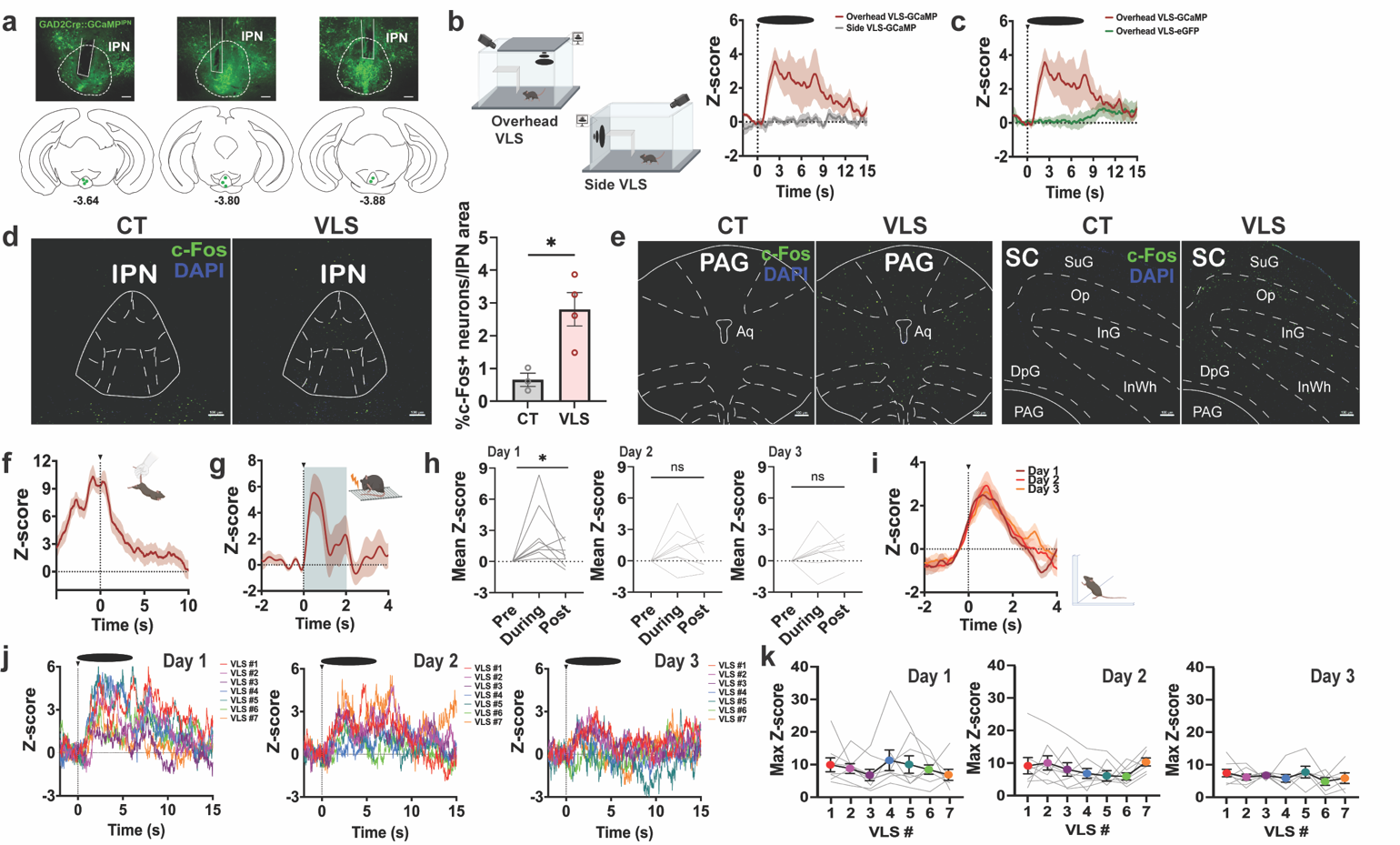
*

## Suppl. Figure 5. IPN GAD2 GCaMP activity responses predict defensive behaviors.

**|** (**a**) Example of speed trace (cm/s) overlapped with IPN *GAD2* fiber photometry signals (dF/F0) across the entire looming sessions, on days 1-3. Initiation of each VLS is indicated by black circle. Correlation between Z-score IPN *GAD2* activity responses on day 1 and (**b**) average speed (cm/s) 10s upon VLS initiation (Pearson’s correlation, r = -0.385, p = 3.672 E-004), (**c**) latency to the nest (Pearson’s correlation, r = 0.523, p = 5.048 E-005) and (**d**) time spent near the walls (%) 30 sec upon VLS initiation (Pearson’s correlation, r = 0.365, p = 0.00658). (n = 26 - 33 VLS events per condition each day, from 8 mice). Data represent mean ± SEM.

*
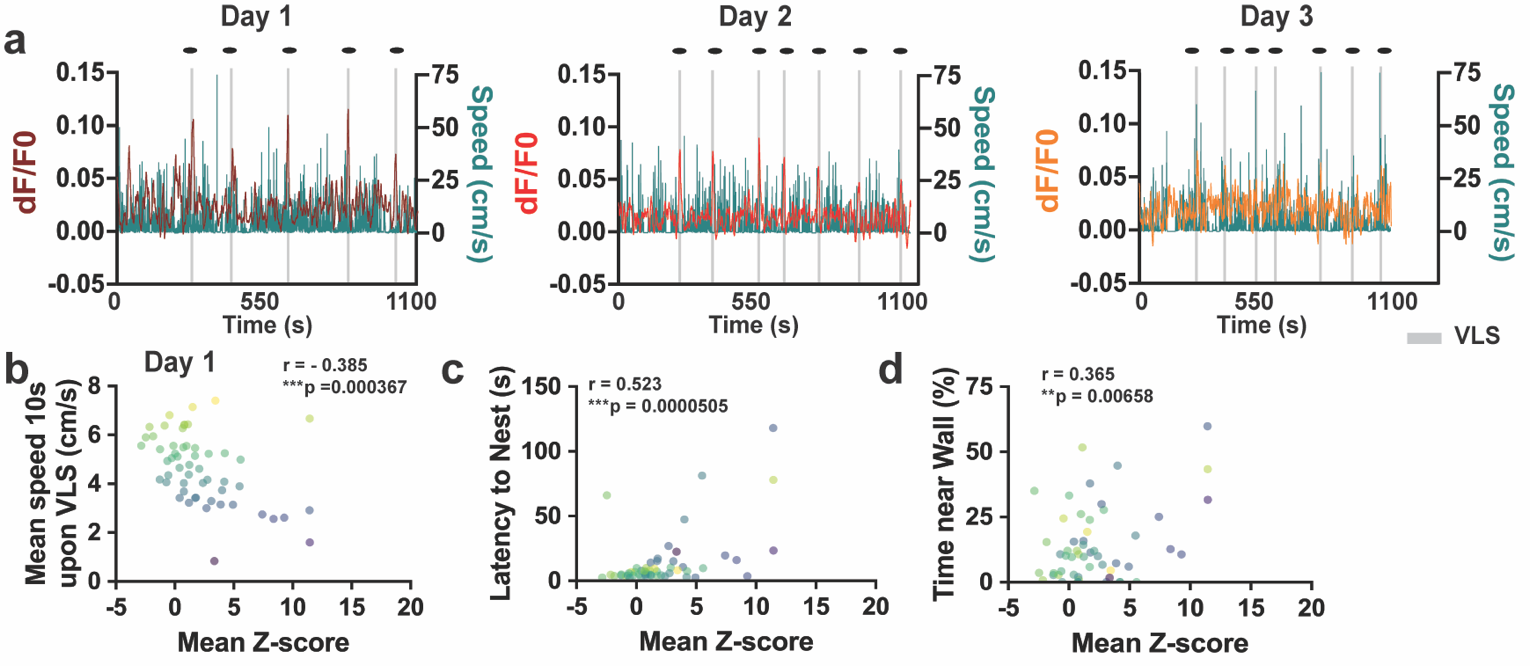
*

## Suppl. Figure 6. Silencing IPN GAD2 neurons increases time nearby safety areas.

**|** (**a**) Representative images and schematics of viral injections and fiber implants, scale bars 100µm. (**b**) Latency to nest (s) relative to VLS, across 3 days in IPN *GAD2* eGFP and NpHR animals. Two-way RM ANOVA (day effect: F_(2,32)_ = 1.517, *P* = 0.2351; treatment effect: F_(1,16)_ = 0.8876, *P* = 0.3601; interaction: F_(2,32)_ = 0.2180, *P* = 0.8053). (**c**) Traces of time spent in the safe zone (%) relative to VLS, across 3 days in IPN *GAD2* eGFP and NpHR animals. (**d**) Quantification of time spent in the safe zone 30 sec upon VLS in (c). Two-way RM ANOVA (day effect: F_(2,32)_ = 1.077, *P* = 0.3515; treatment effect: F_(1,16)_ = 2.365, *P* = 0.1436; interaction: F_(2,32)_ = 3.857, *P* = 0.0316), Tukey’s multiple comparisons *p<0.05. (**e**) Traces of time spent near the wall (%) relative to VLS, across 3 days in IPN *GAD2* eGFP and NpHR animals. (**f**) Quantification of time spent near the wall (%) 30 sec upon VLS in (e). Two-way RM ANOVA (day effect: F_(2,32)_ = 7.239, *P* = 0.0055; treatment effect: F_(1,16)_ = 0.1373, *P* = 0.7158; interaction: F_(2,32)_ = 0.1158, *P* = 0.8911), day effect $$ p < 0.01. (**g**) Traces of time spent in the trigger zone (%) relative to VLS, across 3 days in IPN *GAD2* eGFP and NpHR animals. (**h**) Quantification of time spent in the trigger area (%) 30 sec upon VLS in (g). Two-way RM ANOVA (day effect: F_(2,32)_ = 2.830, *P* = 0.0785; treatment effect: F_(1,16)_ = 0.2695, *P* = 0.6108; interaction: F_(2,32)_ = 0.5648, *P* = 0.5740). (n = 6 eGFP and 12 NpHR3.0 mice). Data represent mean ± SEM.


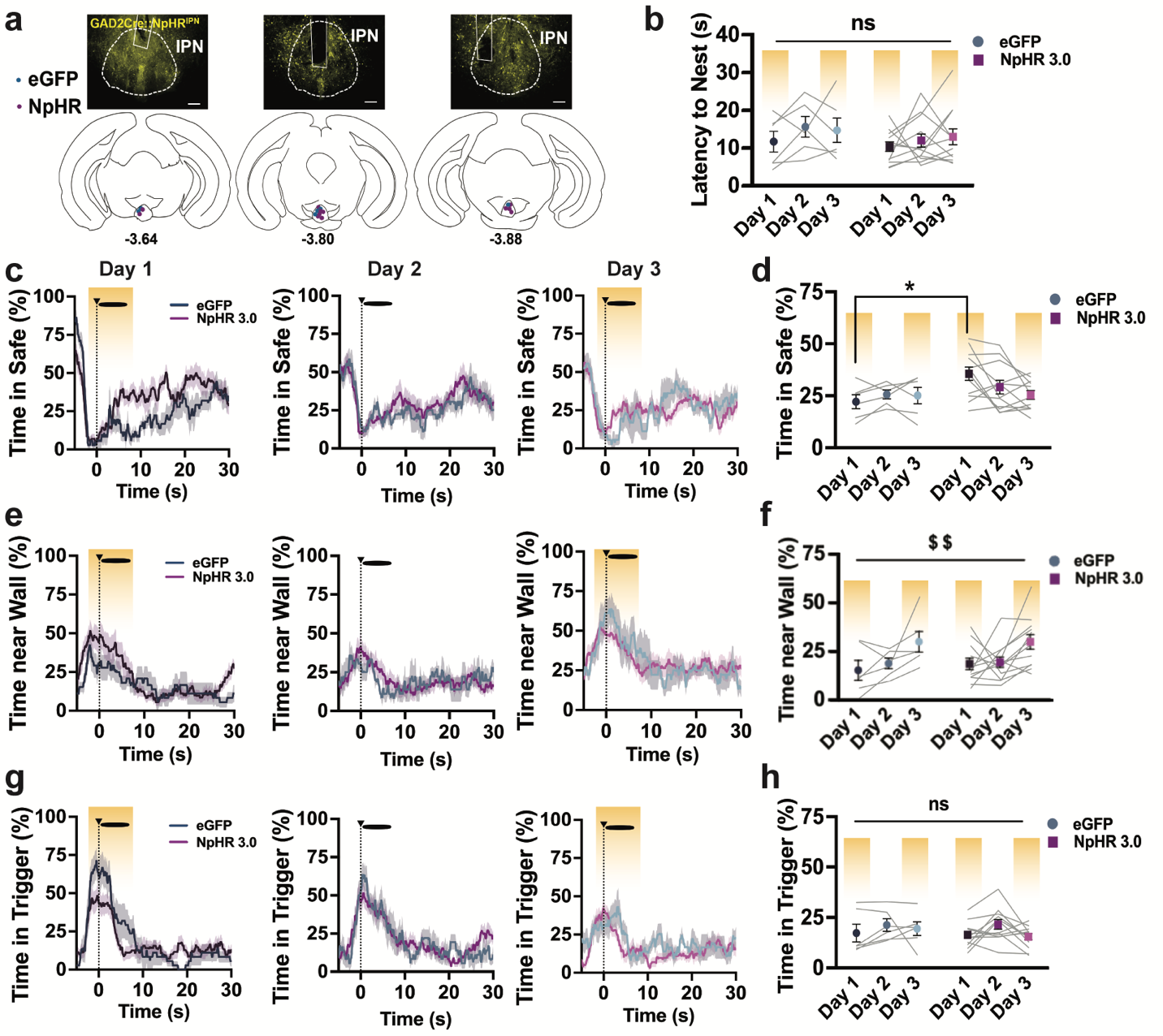


## Suppl. Figure 7. PCA analysis on IPN inhibition behavioral data.

**|** Principal Component Analysis (PCA) was applied to all behavioral variables of VLS responses in GAD2 mice expressing control and NpHR3.0 in the IPN, across three repeated VLS days. The data were reshaped to preserve subject identities for between-group comparisons or identities of behavioral variables. A color code was used to indicate clustered variables across groups and days.

*
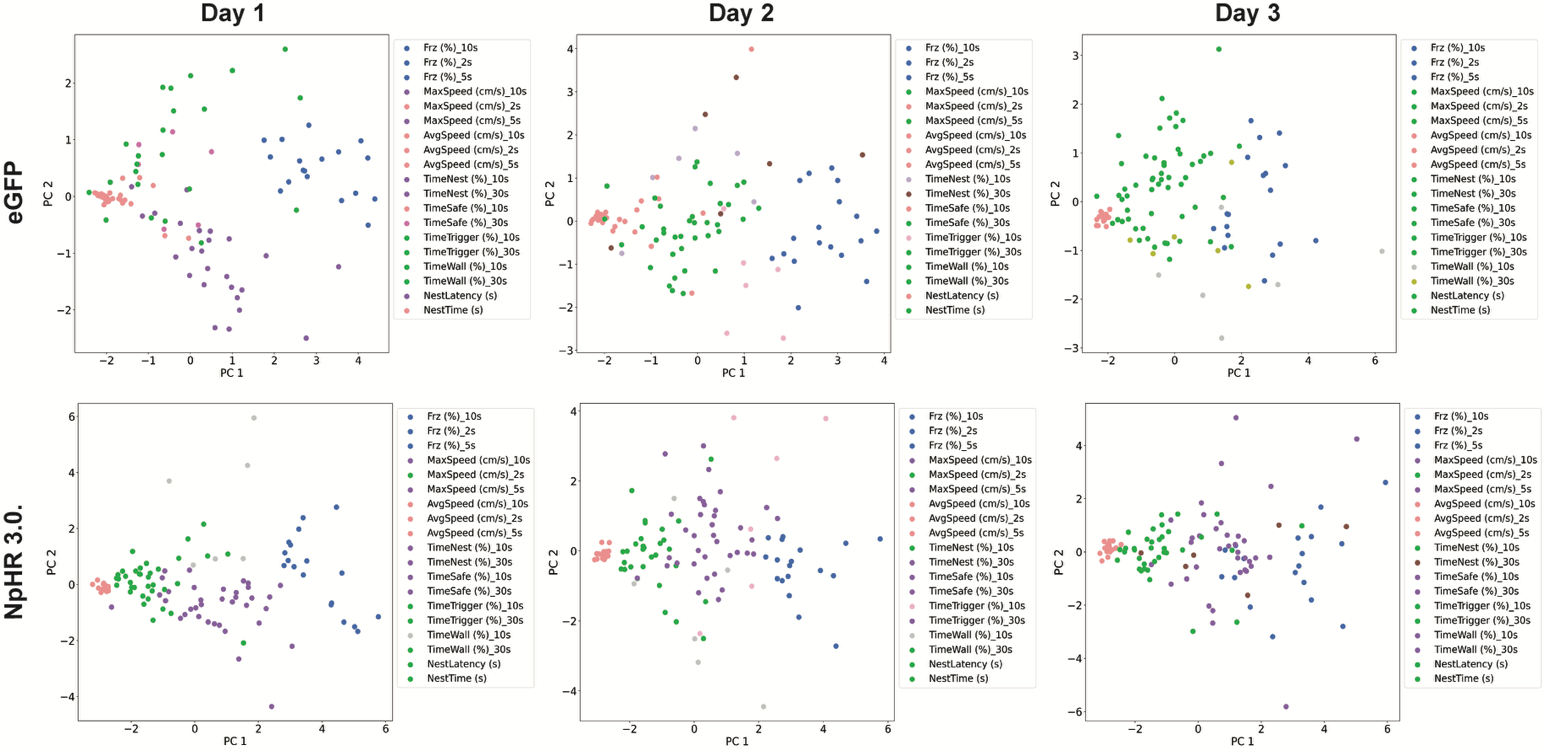
*

## Suppl. Figure 8. Photostimulation of IPN GAD2 neurons is not sufficient to trigger defensive behaviors but impairs threat adaptation.

**|** (**a**) Schematic and representative image of ChR2 injection and fiber placement in the IPN in *GAD2 Cre* mice. Scale bar 100µm. (**b**) Representative images and schematics of viral injections and fiber implants, scale bars 100µm. (**c**) Schematic representation of IPN *GAD2* neuronal photostimulation (blue, 473 nm, 20 Hz, 12 ms pulse, 3s) on day 1 and day 3. (**d**) Latency to nest (s) relative to light photostimulation, across 3 days in IPN *GAD2* mCherry and ChR2 animals. Two-way RM ANOVA (day effect: F_(2,40)_ = 0.8774, *P* = 0.4229; treatment effect: F_(1,20)_ = 0.2504, *P* = 0.6222; interaction: F_(2,40)_ = 0.7381, *P* = 0.4844). (**e**) Traces of time spent inside the nest (%) relative to light photostimulation (t = 0), across 3 days in IPN *GAD2* mCherry and ChR2 animals. (**f**) Quantification of time in nest (s) after photostimulation across days. Two-way RM ANOVA (day effect: F_(2,40)_ = 10.00, *P* = 0.0003; treatment effect: F_(1,20)_ = 0.07145, *P* = 0.7920; interaction: F_(2,40)_ = 0.1960, *P* = 0.8228). (n = 10 mCherry and 12 ChR2 mice). (**g**) Schematic representation of IPN *GAD2* neuronal photostimulation (blue, 473 nm, 20 Hz, 12 ms pulse) paired with VLS presentations, on day 2 and day 3 (blue shadow). (**h**) Representative schematics of viral injections and fiber implants. (**i**) Latency to nest (s) relative to VLS, across 3 days in IPN *GAD2* mCherry and ChR2 animals. Two-way RM ANOVA (day effect: F_(2,34)_ = 0.1601, *P* = 0.8509; treatment effect: F_(1,17)_ = 3.809, *P* = 0.067; interaction: F_(2,34)_ = 0.1740, *P* = 0.8411). (**j**) Traces of time spent inside the nest (%) relative to VLS, across 3 days in IPN *GAD2* mCherry and ChR2 animals. (**k**) Quantification of time in nest (s) after VLS across days. Two-way RM ANOVA (day effect: F_(2,34)_ = 2.760, *P* = 0.1091; treatment effect: F_(1,17)_ = 3.099, *P* = 0.0963; interaction: F_(2,34)_ = 1.999, *P* = 0.1511). (n = 11 mCherry and 8 ChR2 mice). Data represent mean ± SEM.

*
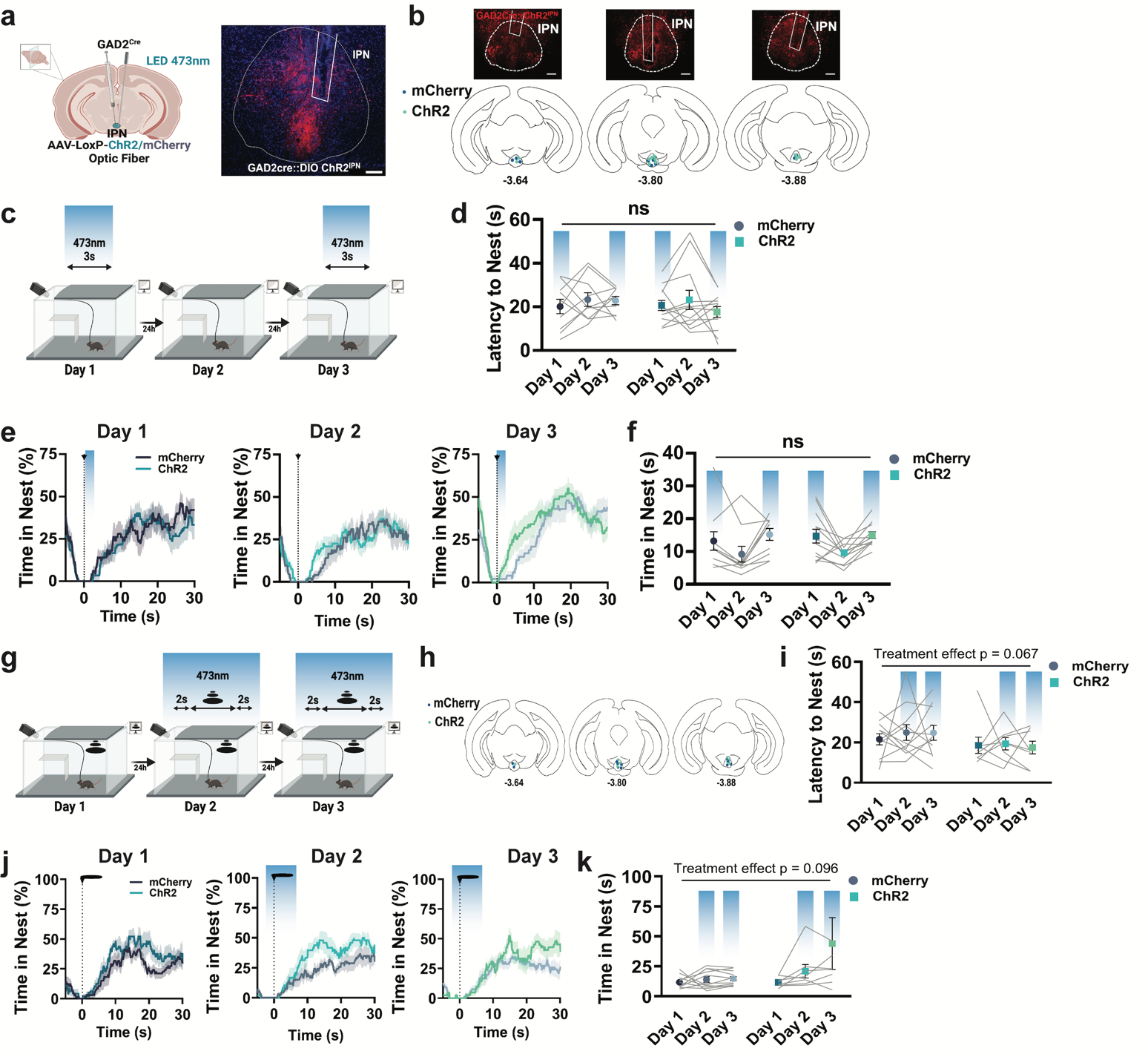
*

## Suppl. Figure 9. IPN🡪LDTg GAD2 GCaMP activity responses predict defensive behaviors.

**|** (**a**) Representative images and schematics of viral injections and fiber implants, scale bars 100µm. (**b**) Mean Z-score values of IPN🡪LDTg GAD2 activity pre (-1 to 0 sec), during (+3 to +4 sec) and post (+13 to +14 sec) VLS events, across day 1 (RM ANOVA, time effect: F_(1.593, 9.557)_ = 22.97, *P* = 0.0003), day 2 (RM ANOVA, time effect: F_(1.158, 6.946)_ = 24.92, *P* = 0.0013) and day 3 (RM ANOVA, time effect: F_(1.693, 10.16)_ = 1.333, *P* = 0.3001). Turkey’s multiple comparisons, **p<0.01. (n = 7 mice). (**c**) Z-score traces of IPN🡪LDTg *GAD2* neuronal activity across VLS events within a session, for 3 consecutive days. (**d**) Maximum Z-score values of IPN🡪LDTg *GAD2* neuronal activity levels across VLS events on day 1 (REML, time effect: F_(2.266,12.08_) = 0.8133, *P* = 0.4802), day 2 (REML, time effect: F_(2.753, 14.22)_ = 0.5369, *P* = 0.6502) and day 3 (REML, time effect: F _(2.115, 10.93)_ = 1.056, *P* = 0.3847). (n = 4 – 7 VLS events/day, from n = 7 mice). Correlation between Z-score IPN🡪LDTg *GAD2* activity responses on day 1 and (**e**) average speed (cm/s) 10s upon VLS initiation (Pearson’s correlation, r = -0.342, p = 0.02129), (**f**) latency to the nest (Pearson’s correlation, r = 0.0812, p = 0.6046) and (**g**) time spent near the walls (%) 30 sec upon VLS initiation (Pearson’s correlation, r = 0.330, p = 0.02681). (n = 13 - 33 VLS events per condition, from 7 mice). Data represent mean ± SEM.

*
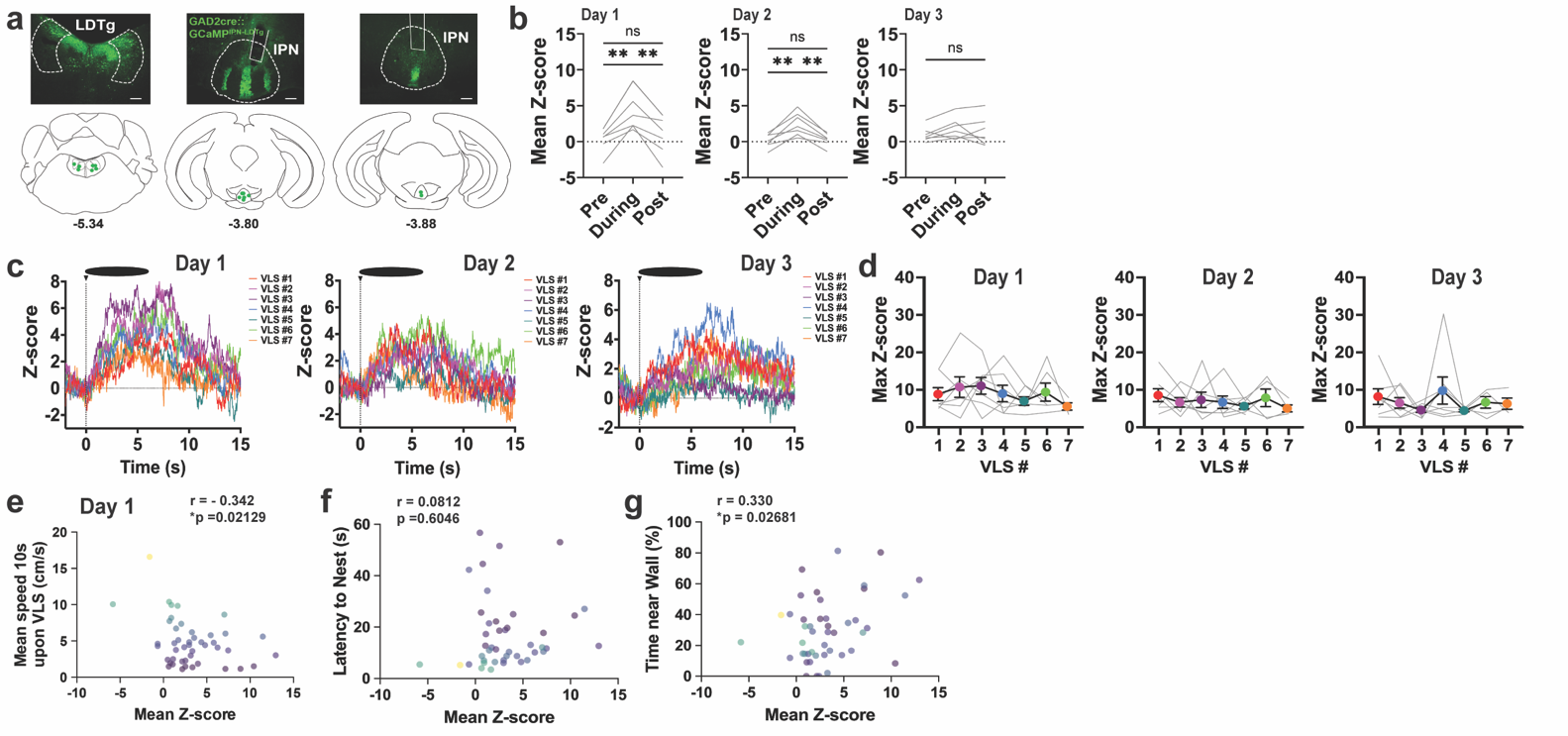
*

## Suppl. Figure 10. Silencing the IPN🡪LDTg GAD2 circuit does not affect additional defensive strategies.

**|** (**a**) Representative images and schematics of viral injections and fiber implants, scale bars 100µm. (**b**) Latency to nest (s) relative to VLS, across 3 days in IPN🡪LDTg *GAD2* eGFP and NpHR animals. Two-way RM ANOVA (day effect: F_(2,60)_ = 0.9975, *P* = 0.3678; treatment effect: F_(1,30)_ = 2.143, *P* = 0.1536; interaction: F_(2,60)_ = 1.788, *P* = 0.1761). (**c**) Traces of time spent in the safe zone (%) relative to VLS, across 3 days in IPN🡪LDTg *GAD2* eGFP and NpHR animals. (**d**) Quantification of time spent in the safe zone 30 sec upon VLS in (c). Two-way RM ANOVA (day effect: F_(2,60)_ = 6.537, *P* = 0.0040; treatment effect: F_(1,30)_ = 0.05379, *P* = 0.8182; interaction: F_(2,60)_ = 2.287, *P* = 0.1104), day effect $$ p < 0.01. (**e**) Traces of time spent near the wall (%) relative to VLS, across 3 days in IPN🡪LDTg *GAD2* eGFP and NpHR animals. (**f**) Quantification of time spent near the wall (%) 30 sec upon VLS in (e). Two-way RM ANOVA (day effect: F_(2,60)_ = 2.106, *P* = 0.1352; treatment effect: F_(1,30)_ = 0.3112, *P* = 0.5811; interaction: F_(2,60)_ = 1.581, *P* = 0.2141). (**g**) Traces of time spent in the trigger zone (%) relative to VLS, across 3 days in IPN🡪LDTg *GAD2* eGFP and NpHR animals. (**h**) Quantification of time spent in the trigger area (%) 30 sec upon VLS in (g). Two-way RM ANOVA (day effect: F_(2,60)_ = 0.09885, *P* = 0.9024; treatment effect: F_(1,30)_ = 0.4902, *P* = 0.4893; interaction: F_(2,60)_ = 0.8449, *P* = 0.4346). (n = 15 eGFP and 17 NpHR3.0 mice). Data represent mean ± SEM.

*
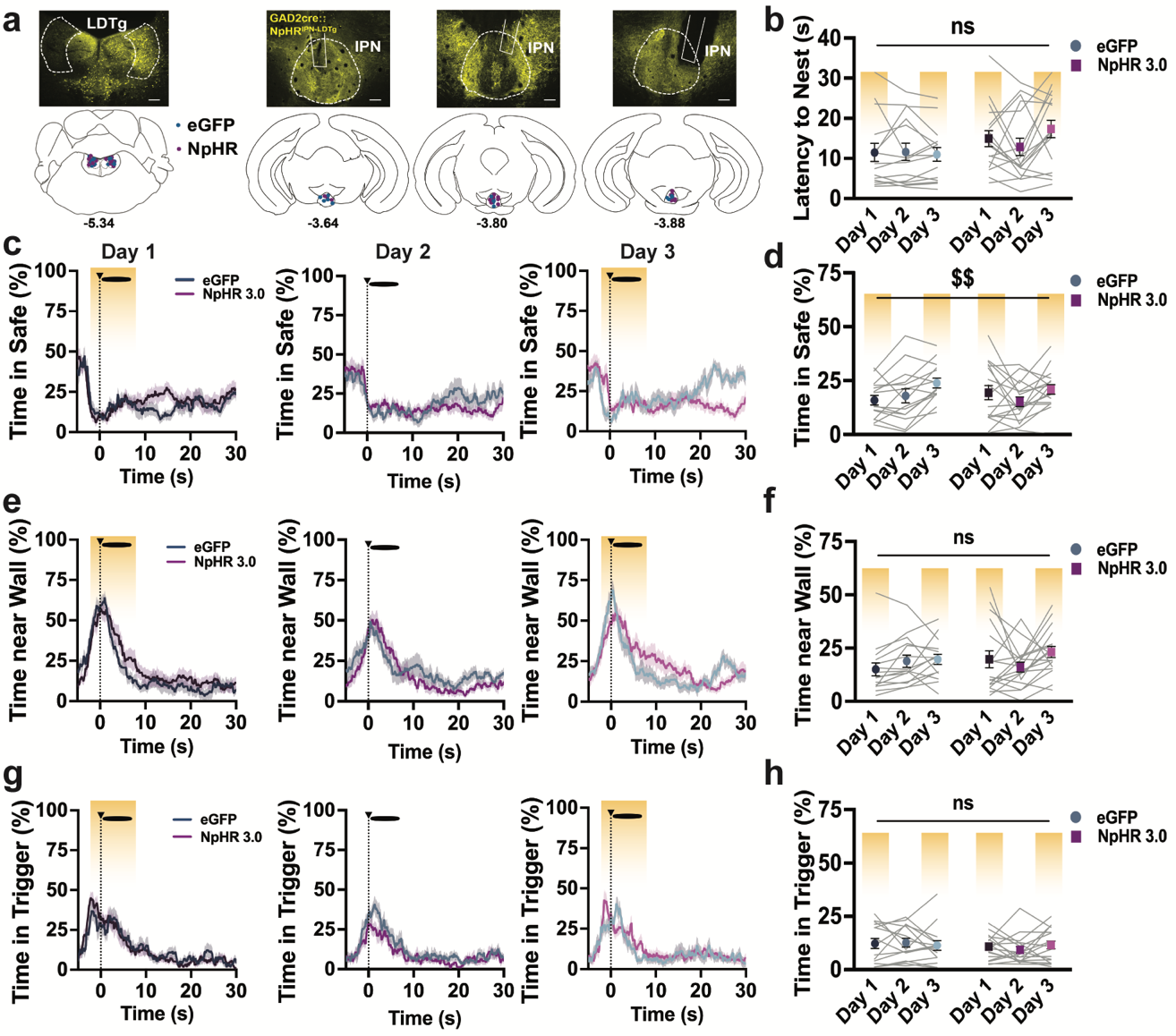
*

## Suppl. Figure 11. IPN Sst+ GCaMP activity responses predict threat and avoidance cues.

**|** (**a**) Representative images of rostro-caudal distribution of immunolabelled Sst^+^ neurons in the IPN. Scale bar 100μm. (**b**) Schematic of viral injection used in Sst Cre mice, with viral injection in the IPN and projections in the LDTg. Scale bars 1000μm. Magnified inset showing IPN axons innervating the LDTg area (white arrows). Scale bar 100μm. (**c**) Schematic of retroviral injection used in Sst Cre mice, with viral injection in the LDTg and cell bodies retrolabelled in the IPN (white arrows). Scale bar 100 and 200μm. (**d**) Representative image of GCaMP expression in the IPN (green) of Sst immunolabelled with Sst staining (red). Nuclei are counterstained with DAPI (blue). Scale bar 50μm. (**e**) Representative images and schematics of viral injections and fiber implants, scale bars 100µm. (**f**) Mean Z-score values of IPN Sst neuronal activity pre (-1 to 0 sec), during (+3 to +4 sec) and post (+13 to +14 sec) VLS events, across day 1 (RM ANOVA, time effect: F_(1.048, 4.191)_ = 4.255, *P* = 0.1046), day 2 (RM ANOVA, time effect: F_(1.057, 4.230)_ = 3.549, *P* = 0.1287) and day 3 (RM ANOVA, time effect: F_(1.345, 5.378)_ = 12.71, *P* = 0.0118). Turkey’s multiple comparisons, *p<0.05. (n = 5 mice). IPN Sst^+^ neuronal activity responses upon a tail lift (**g**) or a foot shock (**h**). Correlation between Z-score IPN Sst^+^ activity responses upon nest entry on day 1 and (**i**) average speed (cm/s) 10s upon VLS initiation (Pearson’s correlation, r = 0.3909, p = 0.0482), (**j**) time in nest (Pearson’s correlation, r = 0.4501, p = 0.0210), (**k**) latency to the nest (Pearson’s correlation, r = -0.3815, p = 0.0544) and (**l**) time spent near the walls (%) 30 sec upon VLS initiation (Pearson’s correlation, r = -0.4148, p = 0.0391). (n = 10 - 15 VLS events per condition each day, from 5 mice). Data represent mean ± SEM.

*
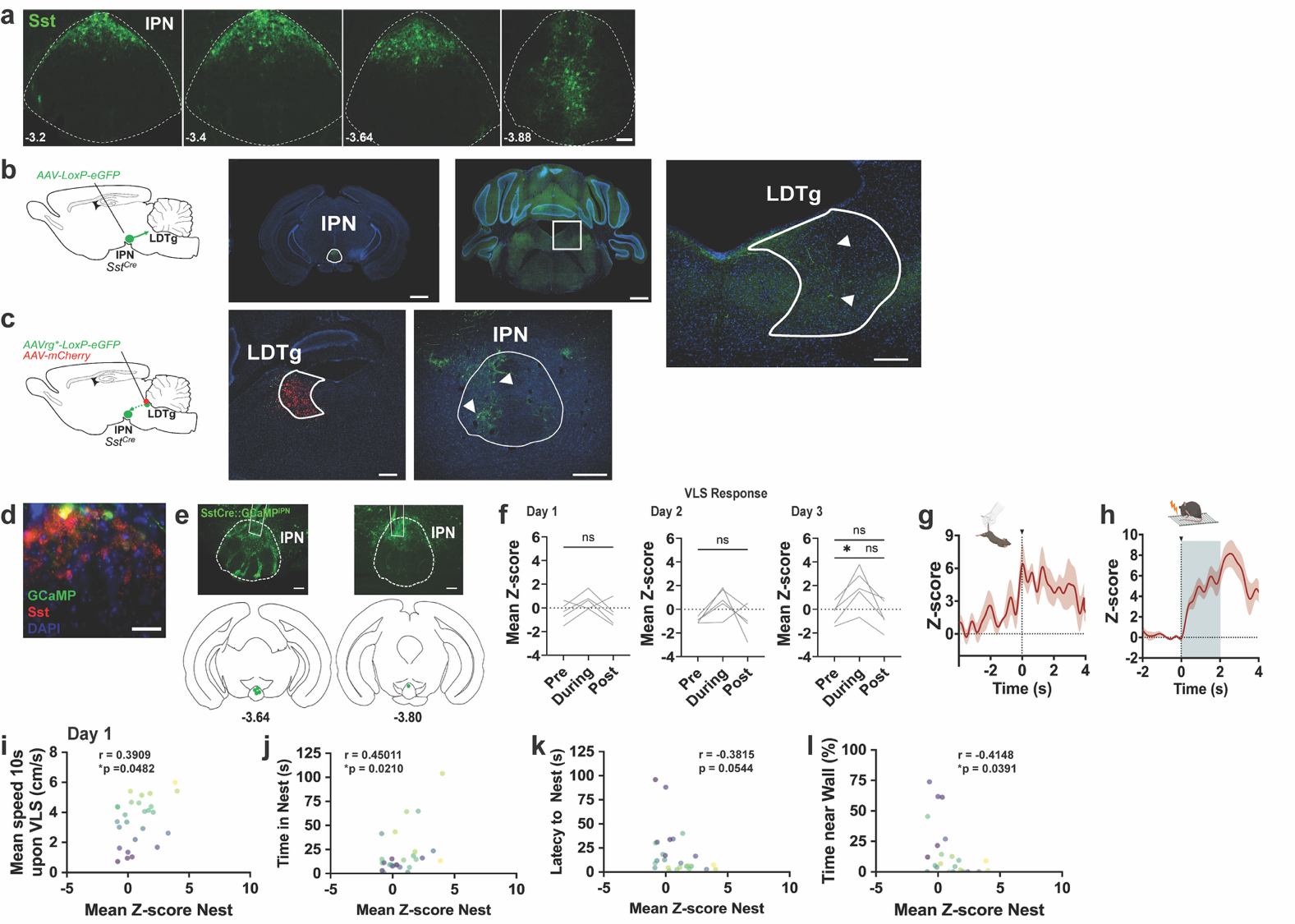
*

## Suppl. Figure 12. Genetic ablation of IPN Sst+ neurons reduces avoidance behaviors.

**|** (**a**) Representative images and schematics of viral injections in control and IPN Sst ablated animals, scale bars 100µm. (**b**) Quantification of Sst+ cells per IPN slice in control and taCasp3-TEVp animals. Unpaired t-test (t_(21)_ = 22.45, *P* < 0.0001). (**c**) Traces of freezing time (%) relative to VLS (t = 0), across 3 days in IPN Sst mCherry and taCasp3-TEVp animals. (**d**) Quantification of freezing time (%) 2 sec upon VLS in (c). Two-way RM ANOVA (day effect: F_(2,38)_ = 16.16, *P* < 0.0001; treatment effect: F_(1,19)_ = 1.694, *P* = 0.2086; interaction: F_(2,38)_ = 0.0402, *P* = 0.9606), day effect $$$ p< 0.001. (**e**) Traces of speed (cm/s) relative to VLS (t = 0), across 3 days in IPN Sst mCherry and taCasp3-TEVp animals. (**f**) Quantification of max speed (cm/s) 2 sec upon VLS in (e). Two-way RM ANOVA (day effect: F_(2,38)_ = 1.471, *P* = 0.2431; treatment effect: F_(1,19)_ = 0.1024, *P* = 0.7525; interaction: F_(2,38)_ = 0.0968, *P* = 0.9080). (**g**) Traces of time spent in the safe zone (%) relative to VLS, across 3 days in IPN Sst mCherry and taCasp3-TEVp animals. (**h**) Quantification of time spent in the safe zone 30 sec upon VLS in (g). Two-way RM ANOVA (day effect: F_(2,38)_ = 1.516, *P* = 0.2352; treatment effect: F_(1,19)_ = 7.176, *P* = 0.0149; interaction: F_(2,38)_ = 0.1821, *P* = 0.8342), treatment effect # p < 0.05. (**i**) Traces of time spent near the wall (%) relative to VLS, across 3 days in IPN Sst mCherry and taCasp3-TEVp animals. (**j**) Quantification of time spent near the wall (%) 30 sec upon VLS in (i). Two-way RM ANOVA (day effect: F_(2,38)_ = 3.161, *P* = 0.0702; treatment effect: F_(1,19)_ = 3.709, *P* = 0.0692; interaction: F_(2,38)_ = 0.3802, *P* = 0.6863). (**k**) Time spent in the open arms of the elevated plus maze (EPM) in IPN Sst mCherry and taCasp3-TEVp animals. Unpaired t test (t_(22)_ = 2.156, *P* = 0.0423). (**l**) Number of entries in the closed arms of the EPM. Unpaired t test (t_(22)_ = 0.310, *P* = 0.7592). (**m**) Time spent in the center of the open field in IPN Sst mCherry and taCasp3-TEVp animals. Unpaired t test (t_(22)_ = 2.22, *P* = 0.037). (**n**) Total distance travelled in the open field. Unpaired t test (t_(22)_ = 0.4099, *P* = 0.6859). * p< 0.05. (n = 11 mCherry and 13 taCasp3-TEVp mice). Data represent mean ± SEM.

*
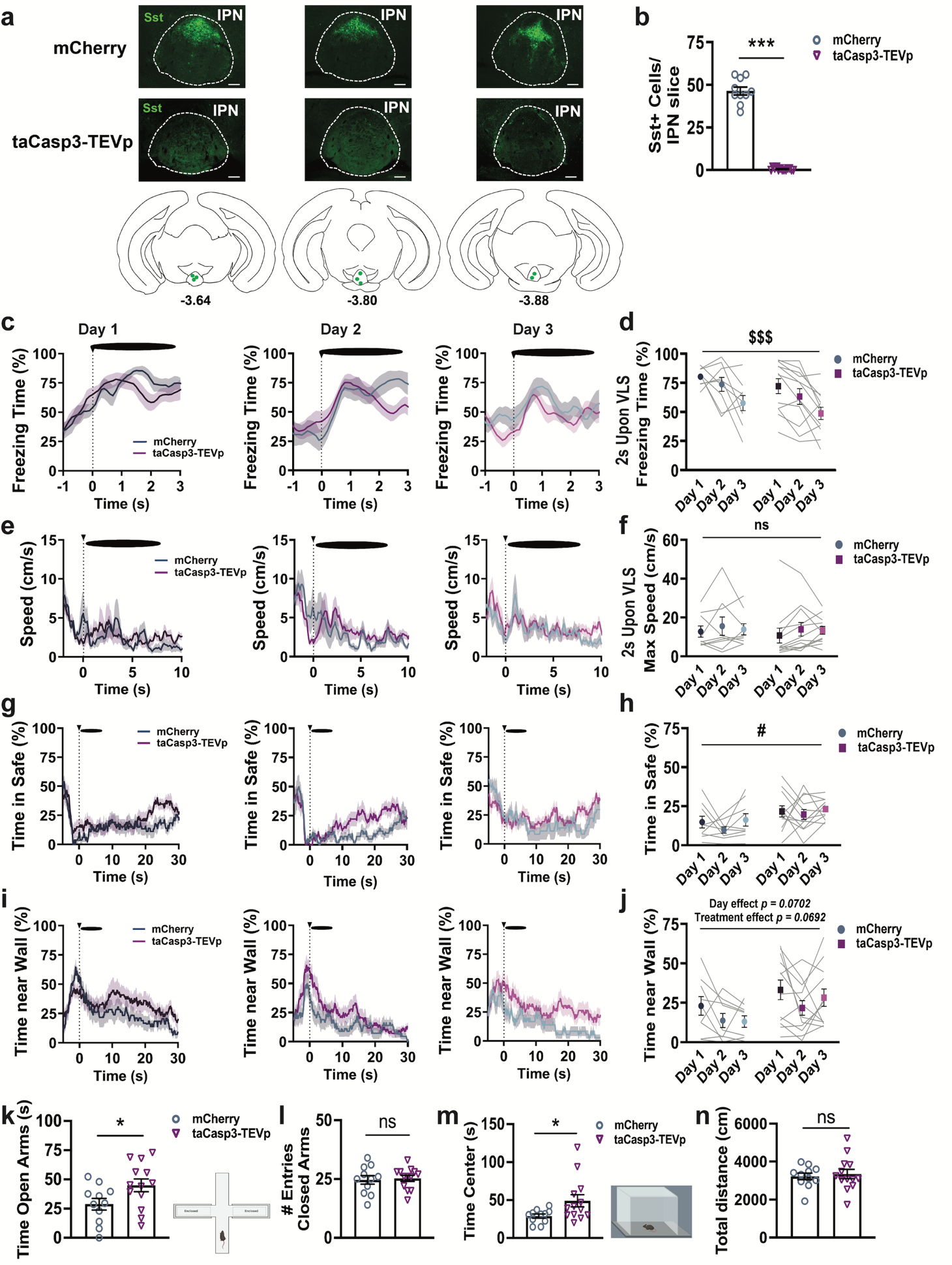
*

## Suppl. Table 1.

Correlation analysis reporting Pearson’s correlation coefficients on day 1. (n = 189 – 192 VLS events/day, from n = 31 mice).

## Suppl. Table 2.

Correlation analysis reporting Pearson’s correlation coefficients on day 2. (n = 189 – 192 VLS events/day, from n = 31 mice).

## Suppl. Table 3.

Correlation analysis reporting Pearson’s correlation coefficients on day 3. (n = 189 – 192 VLS events/day, from n = 31 mice).

## Suppl. Table 4.

Correlation analysis reporting Pearson’s correlation *p* values on day 1. (n = 189 – 192 VLS events/day, from n = 31 mice).

## Suppl. Table 5.

Correlation analysis reporting Pearson’s correlation *p* values on day 2. (n = 189 – 192 VLS events/day, from n = 31 mice).

## Suppl. Table 6.

Correlation analysis reporting Pearson’s correlation *p* values on day 3. (n = 189 – 192 VLS events/day, from n = 31 mice).
